# Impact of bacteria motility in the encounter rates with bacteriophage in mucus

**DOI:** 10.1038/s41598-019-52794-2

**Published:** 2019-11-11

**Authors:** Kevin L. Joiner, Arlette Baljon, Jeremy Barr, Forest Rohwer, Antoni Luque

**Affiliations:** 1 Computational Science Research Center, San Diego State University, San Diego, CA 92182 USA; 2 Naval Information Warfare Center, San Diego, CA 92152 USA; 3 Department of Physics, San Diego State University, San Diego, CA 92182 USA; 4 School of Biological Sciences, Monash University, Clayton, VIC 3800 Australia; 5 Department of Biology, San Diego State University, San Diego, CA 92182 USA; 6 Department of Mathematics and Statistics, San Diego State University, San Diego, CA 92182 USA; 7 Viral Information Institute, San Diego State University, San Diego, CA 92182 USA

**Keywords:** Ecological modelling, Computational science

## Abstract

Bacteriophages—or phages—are viruses that infect bacteria and are present in large concentrations in the mucosa that cover the internal organs of animals. Immunoglobulin (Ig) domains on the phage surface interact with mucin molecules, and this has been attributed to an increase in the encounter rates of phage with bacteria in mucus. However, the physical mechanism behind this phenomenon remains unclear. A continuous time random walk (CTRW) model simulating the diffusion due to mucin-T4 phage interactions was developed and calibrated to empirical data. A Langevin stochastic method for Escherichia coli (*E. coli*) run-and-tumble motility was combined with the phage CTRW model to describe phage-bacteria encounter rates in mucus for different mucus concentrations. Contrary to previous theoretical analyses, the emergent subdiffusion of T4 in mucus did not enhance the encounter rate of T4 against bacteria. Instead, for static *E. coli,* the diffusive T4 mutant lacking Ig domains outperformed the subdiffusive T4 wild type. *E. coli*’s motility dominated the encounter rates with both phage types in mucus. It is proposed, that the local fluid-flow generated by *E. coli*’s motility combined with T4 interacting with mucins may be the mechanism for increasing the encounter rates between the T4 phage and *E. coli* bacteria.

## Introduction

Phages—short for bacteriophages—are viruses that infect bacteria and are the most abundant replicative biological entities on the planet^1,2^, helping to regulate ecosystems and participating in the shunt of nutrients and the control of bacteria populations^3^. These viruses are also abundant in multicellular animals ranging from cnidarians to humans^4^ and, along with their associated bacteria, affect metazoan health^5–8^. Humans host around 10 trillion phages^9^, and about three billion phages penetrate the human body daily^10^. Previous studies have shown how phage help regulate the human microbiota by fighting pathogenic bacteria^4,11–14^, which has important implications to the health industry. However, certain phages that are effective *in vitro* are not consistent in reducing bacteria host concentrations *in vivo*^15,16^, thereby limiting the understanding of how phage control bacteria in their natural environments.

In animals, bacteria and phage heavily colonize the mucus layers that cover internal organs^17,18^. Mucus is mostly composed by large glycoproteins (up to 10^6^–10^9^ Da) called mucins, which self-organize forming a mesh^19^. Mucins are constantly secreted by underlying epithelial cells^20,21^ and are subjected to shear forces that slough off the outermost layers^22^. Secretion maintains a protective mucus layer with a thickness ranging from 10 to 700 *μ*m and depends on species and body location^23–26^. Phages in the human gut contain hypervariable immunoglobulin-like (Ig-like) domains^27^ that resemble antibodies and T-cell receptors^28^, which are displayed in the structural proteins of phages^29,30^. *In-vitro* experiments have shown that the Ig-like domain of the outer capsid protein (hoc) of T4 phage^31^ adhered to mucus, binding weakly to the abundant glycans in mucins^4^; this domain was shown to be prevalent across phages in different environments. The use of a micrfofluidic device (chip) that emulated a mucosal surface^32^ showed that both T4 wild type (T4 wt) and non-adherent (T4Δ*hoc*) phage lacking the hoc domain accumulated to comparable abundance in the mucus chip. However, it was observed that T4 wt reduced bacteria concentration more than 4,000-fold as compared with the T4Δ*hoc*. This motivated a bacteriophage adherence to mucus (BAM) framework, which considers phages as a non-host derived antimicrobial defense^33^ on the mucosal surfaces of meatazoan and assigns subdiffusion as the sole mechanism that drives increased encounter rates of T4 wt with bacteria in mucus^32^. T4 wt phage interacts with mucins and moves subdiffusively; that is, its mean-squared displacement (MSD) increases sublinearly over time: ${\rm{MSD}}(t)=2\,d{D}_{\alpha }{t}^{\alpha }$, where *d* is the number of dimensions, *D*_*α*_ is the generalized diffusion constant, *α* < 1 is the anomalous diffusion exponent^32,34^ and *t* is the time lag. The diffusion exponent of T4 wt depends on the mucin concentration [mucin], and, within the physiological range from 0% to 4% weight per volume (wt/vol), it reaches a minimum value of *α* = 0.82, at ∼1% [mucin]^32^. In contrast, T4Δ*hoc* diffuses regularly (*α* = 1) because it does not adhere with mucins.

The mathematical encounter rate framework for the current BAM model^32^ was based on the work by Golding^35^ and Halford^36^. These authors showed that the average time it takes for a hunter to find its prey is roughly inversely proportional to the hunters diffusion constant assuming the hunter does not move away from the area in which the prey is located. The 2015 BAM model extended this work to one phage and one bacterium. It was proposed that even though the subdiffusive T4 wt phage took an average time longer to encounter a bacterium than the T4Δ*hoc* mutant, it would be more likely to encounter bacteria before moving out of the mucus layer.

In both experiment and literature^32,35,37^ there are several instances which relate subdiffusion to encounter rates that suggest improvement to the 2015 BAM model. First, during experiments, Barr *et al*. observed that due to the turnover dynamics of the mucus layer, both T4 wt and T4Δ*hoc* had similar accumulation and prolonged existence in mucus despite their polarizing diffusive characteristics^32^. Secondly, several previous studies^35,37^ have found anomalous search strategies result in lower encounter rates even if the probability to find the target is increased. Thus, in the current BAM framework there appears to be a broad theoretical gap linking phage subdiffusion to an increased frequency of bacterial encounters in mucus. Finding an appropriate mechanism, either subdiffusion or otherwise, remains to be an interesting and open problem to explore.

In this work, as a follow up to Barr *et al*.^32^, a close examination into the active role that bacteria play in influencing encounter rates with phage is conducted. It is shown that bacteria motility plays a vital role in influencing their encounters with phage. To accomplish this, a computational framework was used to simulate the motion of bacteria and phage, capturing the effects of mucus implicitly. It is demonstrated that a phage search strategy interrupted by sticking times is not more effective than Brownian motion in finding a motile or static bacterium. Specifically, when the bacterium was motile, the encounter rates were independent of phage diffusion. It is hypothesized that bacterial motility and the fluid flow generated by bacteria swimming, combined with BAM attachment increases the infectivity of T4 wt versus T4Δ*hoc*. In this way, the mucus may act as a coupling mechanism between the microbes via their motive dynamics to increase encounter rates. During the development of the methodology, the study addresses and solves several interesting issues for a broad audience in physics and biology, e.g., rheological probing with phage, how to calibrate a subdiffusive system with empirical data and general expressions for encounter rates were derived that include both diffusion and subdiffusion.

## Methods

**Figure 1:**
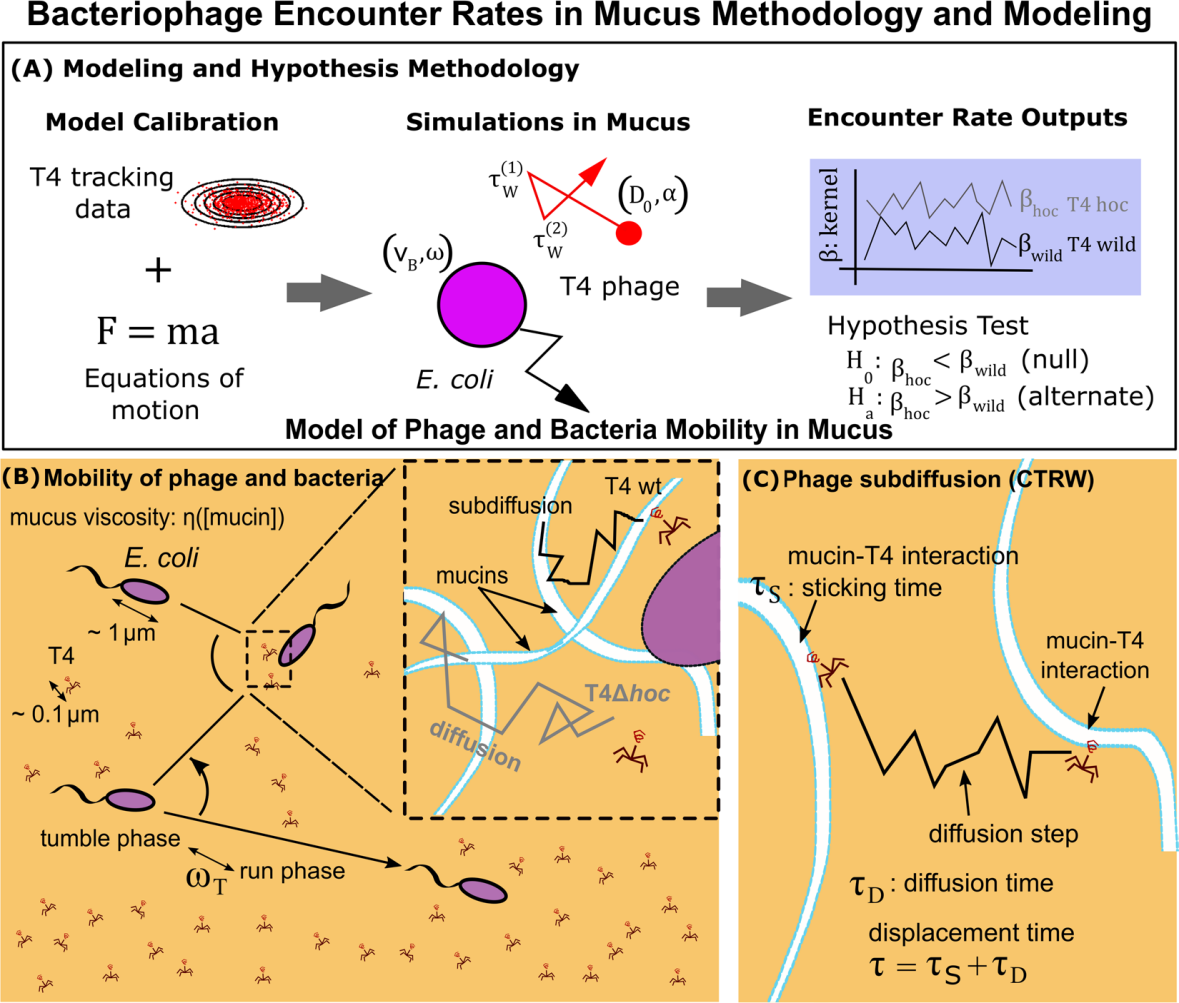
Bacteriophage Encounter Rates in Mucus Methodology and Modeling. (**A**) Calibrated models of phage and bacteria in mucus is used to evaluate the effect of subdiffusion on encounter rates. (**B**) The mucus layer was homogeneous, and its viscosity $\eta$ depended on the mucin concentration $[{\rm{mucin}}]$. *E. coli* propelled through mucus in a straight line in the *run phase*, and reoriented randomly in the *tumble phase*. The tumble rate $({\omega }_{T})$ was the frequency between the two phases. In the inset, phage T4 wildtype (wt) displayed subdiffusion due to its interaction with mucins, while T4Δ*hoc* displayed regular diffusion (no adherence to mucin). (**C**) The subdiffusion was generated using a continuous time random walk (CTRW) model; the total displacement time (*τ*) was composed by the sticking time (${\tau }_{S}$) due to the interaction of T4 and mucins and the diffusion time (${\tau }_{D}$) due to the diffusion of T4 between mucins.

Langevin models were incorporated to simulate T4 and Escherichia coli (*E. coli*) microbes in a homogeneous mucus layer with a mucin concentration dependent viscosity, ${\eta }_{M}$, (Fig. 1A). The models were calibrated using empirical data, which minimized parameters and simplified computational complexity without sacrificing accuracy (Fig. 1A). Diffusion constants and exponents for both T4 wt and T4Δ*hoc* (Table 1), previously obtained via high-speed multiple particle tracking and reported in Barr *et al*.^32^, were used for calibrating the phage models over different values of mucin concentration (see Results: Phage and Bacteria Model Calibration).

**Table 1:** Mucin concentration [mucin] in (% w/v), anomalous exponent, *α*, generalized diffusion constants *D*_*α*_ (*μ*m^2^/s^*α*^) and standard diffusion constants *D* (*μ*m^2^/s) obtained experimentally for T4 wild type and T4Δ*hoc* phages respectively^32^. Values are averages ± SD (standard deviation).

[mucin]	T4 wild type		T4Δ*hoc*	
	*α*	*D*_*α*_	*α*	*D*
0	1.02 ± 0.02	3.84 ± 0.17	0.99 ± 0.03	3.64 ± 0.24
0.2	0.93 ± 0.02	2.38 ± 0.17	0.99 ± 0.04	3.14 ± 0.27
0.6	0.82 ± 0.02	1.18 ± 0.12	1.01 ± 0.02	2.63 ± 0.11
1	0.82 ± 0.01	1.03 ± 0.07	1.02 ± 0.02	2.54 ± 0.14
2	0.91 ± 0.02	0.90 ± 0.07	0.99 ± 0.02	1.37 ± 0.07
4	0.86 ± 0.02	0.42 ± 0.04	0.89 ± 0.02	0.74 ± 0.04

### Phage diffusion and subdiffusion in mucus

Phage diffusion in mucus was modeled in three dimensions by the Langevin equation:

1 $$\mathop{\underbrace{{m}_{P}{\dot{{\boldsymbol{v}}}}_{P}(t)}}\limits_{{\rm{inertia}}}=-\,\mathop{\underbrace{{\gamma }_{P}{{\boldsymbol{v}}}_{P}(t)}}\limits_{{\rm{friction}}}+\mathop{\underbrace{\sqrt{2{\gamma }_{P}kT}{\boldsymbol{W}}(t)}}\limits_{{\rm{thermal}}\,{\rm{noise}}}.$$

The vector ${{\boldsymbol{v}}}_{P}(t)=\langle {v}_{x}(t),{v}_{y}(t),{v}_{z}(t)\rangle$ represented the phage velocity, the scalars ${m}_{P}\approx 0.1\,{\rm{fg}}$^38^ and ${\gamma }_{P}=6\pi {R}_{P}\,{\eta }_{M}$ represented the phage mass and coefficient of friction respectively. The parameter *R*_*P*_ was the effective phage hydrodynamic radius. Both *R*_*P*_ and ${\eta }_{M}$ where extracted from empirical data (see “Viscosity at different mucus concentrations”). The thermal noise term with Gaussian white noise ***W***(*t*) accounted for buffeting from neighboring fluid molecules, and $kT$ was the thermal energy of the fluid. Besides thermal buffeting and drag, no other hydrodynamics were included in the initial models (see Discussion).

Phage T4 wt subdiffuses due to its adherence with mucins, while T4Δ*hoc* (no mucin adherence) displays regular diffusion (inset of Fig. 1B). Based on microscopy tracks, T4 wt was interpreted as arresting its motion (sticking) during an interaction with mucin molecules, detaching following a statistically longtailed distribution of sticking times (*τ*_*S*_) and diffusing for a time (*τ*_*D*_) until reaching another mucin molecule (Fig. 1C). A framework incorporating continuous time random walks (CTRW) was used to extract the minimum sticking time (*τ*_0_) and diffusion time (*τ*_*D*_) between mucin interactions. Displacement times (*τ*) were generated using a Pareto probability distribution, $\psi (\tau ;\nu ,{\tau }_{0})=\nu /{\tau }_{0}{({\tau }_{0}/\tau )}^{1+\nu }$, which is a fat-tailed distribution known to give rise to subdiffusion^39^. New mathematical expressions were derived to get appropriate displacement times and fit the minimum sticking time (*τ*_0_) and Pareto exponent (*ν*) to empirical data (see results). A concise description of the derivation is provided below. The reader is referred to the supplementary material for further details.

### Phage minimum sticking times and diffusion times

An asymptotic approximation for the mean-squared displacement (MSD) of random walks was derived using established methods in the study of CTRW^40^. The displacement time was decomposed implicitly as a sticking time and a diffusion time, that is, $\tau ={\tau }_{S}+{\tau }_{D}$ (Fig. 1C). The probability of being at position ***x*** at time *t* was built assuming time-space decoupling and independent and identically distributed processes. Fourier and Laplace transforms were combined with an indicator function approach to obtain the mean-square displacement, ${\rm{MSD}}(t)=2dD{\tau }_{D}\langle n(t)\rangle$, where *d* was the number of dimensions, *D* was the standard diffusion constant and *n* was the number of diffusion steps (Fig. 1C) produced in time $t={\sum }_{i=1}^{n}{\tau }_{i}$. The expression for $\langle n(t)\rangle$, the average number of diffusion steps in time *t*, was obtained asymptotically by adapting a method based on incomplete gamma functions expressed as holomorphic functions^41^. This led to the asymptotic expression $\langle n(t)\rangle  \sim sinc(\nu ){(t/{\tau }_{0})}^{\nu }$ where the constraint between the minimum sticking time (*τ*_0_) and the diffusion time (*τ*_*D*_) of one diffusion step was ${\tau }_{0}={\tau }_{D}\,sinc{(\nu )}^{1/\nu }$. A consistency argument was then used to recover the standard diffusion constant in the limit $\nu \to 1$. A system of two equations involving the minimum sticking time and diffusion time in terms of the phage generalized diffusion constant (*D*_*v*_), standard diffusion constant (*D*) and Pareto exponent ($\nu$) were obtained, for which *τ*_0_ and *τ*_*D*_ can be extracted from empirical data to simulate T4 phage in mucus given in the results section and explicitly derived in the supplementary material.

### Viscosity at different mucus concentrations

The momentum relaxation time, ${\tau }_{{\rm{relax}}}=m/\gamma$, is the time scale on which a particle transitions from smooth ballistic behavior to diffusive behavior. It depends on the particles mass (*m*) and coefficient of friction (*γ*) which is related to the particle size and viscosity of the surrounding fluid. The viscosity of mucus (*η*_*M*_) was extracted using the spherical Stokes-Einstein equation, $D=kT/6\pi {\eta }_{M}{R}_{P}$, where *D* was the standard diffusion constant of phage T4Δ*hoc* in 0%, 0.2%, 0.6%, 1%, 2%, and 4% mucin concentration (weight/volume, w/v) as measured in Barr *et al*.^32^ at 37 °C. Given the emprical data, thermal energy was assumed at 37 °C ($kT=4.28$ pN · nm) which approximates typical temperatures (37°–40 °C) in humans and animals^42^. The effective phage hydrodynamic radius, *R*_*P*_, was determined by assuming the 0% mucus viscosity resembles that of water^43^ (${\eta }_{W}=0.69$ mPa · s) at 37 °C. This led to ${R}_{P}=kT/6\pi {\eta }_{W}D=90\,{\rm{nm}}$ which is similar to half the total length of typical T4 phage^31,44^: 103.5 nm (=120 nm (head) + 93 nm (tail) divided by two).

**Figure 2:**
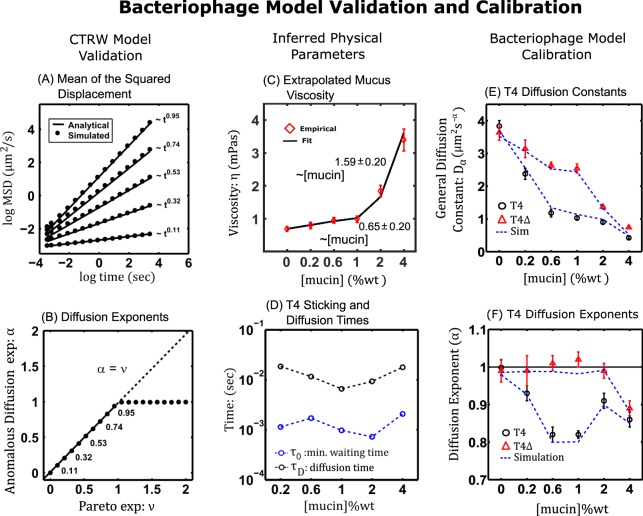
Bacteriophage Model Validation and Calibration. (**A**) Mean square displacement. Plots (log–log) of the simulated data (black circles) is asymptotic to $D\,{t}^{\alpha }/{\tau }_{0}^{\alpha -1}$ (solid lines). (**B**) For $0 < \nu  < 1$, $\alpha =\nu$ (subdiffusion) and $\nu  > 1$, gives $\alpha =1$ (diffusion). (**C**) Mucus viscosity, $(\eta )$, as a power function $\sim {[{\rm{mucin}}]}^{a}$ of mucin concentration. (**D**) Characteristic minimum sticking times (blue circles) and displacement times (black circles) for T4 *wt* across mucin concentrations. (**E,F**) Emergent diffusion characteristics (dashed lines) of simulations compared with empirical T4 wild type (black circles) and T4$\Delta$hoc (red triangles) data.

The mucus viscosity *η*_*M*_ (Table 2) was fitted to two power functions, $${\eta }_{M}=a{[{\rm{mucin}}]}^{b}+{\eta }_{W}$$, where *a* and *b* are fitting parameters, in the regions 0–1% [mucin] and 1–4% [mucin]. In the region 0–1, the viscosity increased sublinearly (Fig. 2C) with an exponent $$b=0.65\pm 0.14$$ where the second digit of *b* is the standard error on its estimate from the linear regression (Table 3). For the region 1–4%, the viscosity increased superlinearly with an exponent $$b=1.59\pm 0.20$$ (Table 4). The crossover scaling of *η*_*M*_ at 1% [mucin] measured here was qualitatively similar to those found in previous rheological studies on the dependence of stomach mucus viscocity on concentration ($${\eta }_{M}\, \sim \,{[{\rm{mucin}}]}^{0.53}\,{\rm{and}}\,{\eta }_{M}\, \sim \,{[{\rm{mucin}}]}^{3.92}$$, with crossover at approximately $$1 \% [{\rm{mucin}}]$$)^45^ and ($${\eta }_{M}\, \sim \,{[{\rm{mucin}}]}^{0.50}$$$${\rm{and}}\,{\eta }_{M}\, \sim \,{[{\rm{mucin}}]}^{1.50}$$, with crossover also at approximately $$1 \% [{\rm{mucin}}]$$)^46^. In this study, the extrapolated mucus viscosities across mucus concentrations were less than an order of magnitude from water implying the relaxation time of the bacteria and phage should be near to that experienced in water: $${\tau }_{{\rm{relax}}}\approx 0.05\,\mu {\rm{s}}$$.

**Table 2:** Mucus viscosity across concentrations. Values are in mPas. Values and errors were extracted using the T4Δ*hoc* diffusion coefficient errors.

Viscosity (*η*)	Mucus Concentration (%wt/vol)					
	0%	0.2%	0.6%	1%	2%	4%
Error (Upper)	0.77	0.90	1.04	1.09	2.01	3.73
Mean	0.69	0.80	0.96	0.99	1.83	3.39
Error (Lower)	0.62	0.70	0.87	0.89	1.66	3.06

**Table 3:** Mucus viscosity regression analysis using the mean values of mucus viscosity.

Viscosity Regression Analysis (0.2–1%)		
Coefficients	Estimate	Std. Error
(Intercept)	−1.12	0.13
*b*	0.65	0.14

The fitting model was $\log ({\eta }_{M}-{\eta }_{W})=b\,\log ([{\rm{mucin}}])+A$. Residual standard error is 0.16 on 1 degree of freedom and a multiple R-squared of 0.92 (on the fitting model).

**Table 4:** Mucus viscosity regression analysis using the mean values of mucus viscosity. The fitting model was $\log ({\eta }_{M}-{\eta }_{W})=b\,\log ([{\rm{mucin}}])+A$. Residual standard error is 0.20 on 1 degree of freedom and a multiple R-squared of 0.98 (on the fitting model).

Viscosity Regression Analysis (1–4%)		
Coefficients	Estimate	Std. Error
(Intercept)	−1.13	0.18
*b*	1.59	0.20

### Bacteria motion in mucus

*E. coli* propels itself through mucus in a straight line during a *run phase*, and reorients randomly in a *tumble phase* at a tumble rate $({\omega }_{T})$ which is the frequency between the two phases^47^. The motion of *E. coli* (Fig. 1B) was modeled using a Langevin equation for which the velocity of the bacterium, ***v***_*B*_, was given by:

2 $$\mathop{\underbrace{{m}_{B}{\dot{{\boldsymbol{v}}}}_{B}(t)}}\limits_{{\rm{inertia}}}=\mathop{\underbrace{{{\boldsymbol{f}}}_{B}(t)}}\limits_{{\rm{flagellar}}\,{\rm{prop}}.}-\mathop{\underbrace{{\gamma }_{B}{{\boldsymbol{v}}}_{B}(t)}}\limits_{{\rm{friction}}}+\mathop{\underbrace{\sqrt{2{\gamma }_{B}kT}{\boldsymbol{W}}(t)}}\limits_{{\rm{thermal}}\,{\rm{noise}}}.$$

Here, *m*_*B*_ was the the mass of *E. coli* ≈1 pg^48^, ***f***_*B*_ the propelling force generated by the rotation of the flagella, and ${\gamma }_{B}=6\pi {R}_{B}\,{\eta }_{M}$ the coefficient of friction for the bacterium.

In three dimensions, $${{\boldsymbol{f}}}_{B}(t)=\langle {f}_{x}(t),{f}_{y}(t),{f}_{z}(t)\rangle $$ and was given by: $${f}_{x}=F(t)\cos \,\lambda (t)\cos \,\theta (t)$$, $${f}_{y}=F(t)\cos \,\lambda (t)$$$$\sin \,\theta (t)$$ and $${f}_{z}=F(t)\sin \,\lambda (t)$$, where *λ* and *θ* were uniform identically independent random numbers between 0 and $$2\pi $$ chosen at run time intervals $${\tau }_{R}$$. In the run phase, with $${v}_{B}=30\,\mu {{\rm{msec}}}^{-1}$$, $$F(t)={v}_{B}$$ and $$F(t)=0$$ during tumbles^47^. The run times were exponentially distributed as $$\Phi ({\tau }_{R})={\omega }_{T}\,\exp (-{\omega }_{T}\,{\tau }_{R})$$^47^. The standard run rate was $${\omega }_{T}=1{\sec }^{-1}$$, giving an average bacteria run time $$\langle {\tau }_{R}\rangle =1/{\omega }_{T}=1{\rm{s}}$$^47,49^, although shorter run times were explored for $${\omega }_{T}=100\,{\sec }^{-1}$$. A static bacterium was also studied with a fixed position, $${{\boldsymbol{v}}}_{B}(t)=0$$, over the course of the simulations.

### Method for bacteria-phage encounter rates

Simulations generated outputs of bacteria–phage encounter rate kernels (*β*) which were defined as the volume searched by a bacterium per unit time. This was later used to test the hypothesis that subdiffusion increases encounter rates (Fig. 1A). To accomplish this, a single bacterium was simulated in a box with volume *V* (bacteria concentration $B=1/V$) with a phage concentration (*P*). The simulation box had dimensions $100\,\mu {\rm{m}}\times 100\,\mu {\rm{m}}\times 100\,\mu {\rm{m}}$ with periodic boundary conditions on all sides. Simulations were initialized with 10^8^ uniformly and randomly distributed T4 wt or T4Δ*hoc* phage particles (equivalent to 1 phage and 1 bacterium simulated 10^8^ times) interacting with mucin. The microbes stick/run timers were initially set to zero and updated during the first simulation step. Once the timers reached zero, the phage or bacterium were displaced/reoriented and the timers were reset to the current time plus the stick/run time. Only interactions between phage and the bacterium were modeled (no intraspecies interactions).

From the mass action principle, the encounter rate kernel is given by $\beta =(1/BP)\,dP/dt=(V/P)\,dP/dt$ [volume · bacteria^−1^ · time^−1^] and was estimated computationally. For this study, adsorption of the phage into the bacterium was instantaneous and no other interactions (i.e., bacteria–phage collisions) were modeled. The adsorption radius, $({R}_{C}={R}_{B}+{R}_{P}=1.1\,\mu {\rm{m}})$, was defined using ${R}_{B}=1\,\mu {\rm{m}}$^47^ and ${R}_{P}=0.1\,\mu {\rm{m}}$ (see “Viscosity at different mucus concentrations”) as the bacterium and phage radii respectively. When a phage was within the adsorption radius from the bacterium an encounter was tallied and the phage was set to an “inactive” state and would not count towards any simulated encounters temporarily. Once the phage was 50 *μ*m away from the bacterium, the phage would be “reactivated” in the simulation. Over the time step, the encounter kernel was calculated as $\beta =({N}_{{\rm{enc}}}V)/({N}_{{\rm{act}}}{\tau }_{D})$, were ${N}_{{\rm{enc}}}$ was the number of encountered phage and ${N}_{{\rm{act}}}$ was the number of active phage. Simulations were visually inspected for clear trends in the emergent encounter kernels which would quickly manifest after $\approx 500$ steps from execution. The simulations were repeated for the range of bacteria motility states shared in the bacteria motion in mucus section above.

### Encounter rate approximation of two motile species

An approximation to the phage–bacteria encounter rate kernel, which extends previous encounter kernel models^50,51^, was derived using a modified diffusion equation and a rest frame centered on the bacterium. The propagator *P* was the probability for a phage to be at position *r* at time *t* and was modeled using the modified diffusion equation in radial coordinates

3 $$\frac{\partial P}{\partial t}=\frac{{\mathfrak{D}}(t)}{{r}^{2}}\frac{\partial }{\partial r}[{r}^{2}\frac{\partial P}{\partial r}],$$

where ${\mathfrak{D}}(t)=(1/2d)\,\partial \langle {r}^{2}\rangle /\partial t$ was the instantaneous diffusion coefficient^52^ of the particles in dimension *d*, and *r* was the radial distance between the bacterium and phage centers. Similar models, using a instantaneous diffusion coefficient, have previously been shown to reproduce the power law dependence of the MSD of anomalously diffusing particles^52,53^. Adopting a restframe centered on the bacterium (located at the origin) where the bacterium was stationary and the phage move in relative motion results in a relative phage MSD $\langle {r}^{2}\rangle$ expressed as

$$\langle {r}^{2}\rangle (t)=\mathop{\underbrace{2d\frac{{v}_{B}^{2}\langle {\tau }_{{\rm{R}}}\rangle }{3}t}}\limits_{{\rm{bacteria}}\,{\rm{MSD}}}+\mathop{\underbrace{2d{D}_{\alpha }{t}^{\alpha }}}\limits_{{\rm{phage}}\,{\rm{MSD}}},$$

which is a superposition of the MSD’s of the bacteria and phage microbes. In $d=3$, taking the derivative of $\langle {r}^{2}\rangle$ with respect to time gives ${\mathfrak{D}}(t)={v}_{B}^{2}\langle {\tau }_{{\rm{R}}}\rangle /3+\alpha {D}_{\alpha }{t}^{\alpha -1}$. The encounter rate problem was defined by Eq. (3) with the boundary condition $P(0,t)=0$ and initial conditions $P(r,0)=0,\,{\rm{if}}\,r\le {R}_{C}$ and $P(r,0)={P}_{\infty },\,{\rm{if}}\,r > {R}_{C}$. Transforming the problem into a 1-D framework using the substitution $v(r,t)=P(r,t)r$ and applying Fourier methods^54^ gave an analytic expression for *P* (see full derivation in the Supplementary Information) that was used to compute the phage flux (encounter rates): ${\mathscr{J}}(t)=\oint {\mathfrak{D}}(t)(\partial P/\partial r)\hat{r}\cdot d\overrightarrow{s}$ evaluated at $r={R}_{C}$ to obtain theoretical approximations to the phage encounter kernel to compare with the simulation results (see Results).

## Results

### Phage continuous time random walks in mucus: an example computation

A first goal of the study was to determine how simulated phage trajectories could be calibrated so they would resemble their laboratory trajectories. The method for deriving equations for the phage minimum sticking times (${\tau }_{0}$) and diffusion times (${\tau }_{D}$) resulted in general expressions for calibrating random walk models to generate simulated trajectories using the Pareto distribution $\psi (\tau ;\nu ,{\tau }_{0})$. Specifically, the constraint between the minimum sticking time and the diffusion time (${\tau }_{D}$) was

4 $${\tau }_{0}={\tau }_{D}\,sinc{(\nu )}^{1/\nu }.$$

This led to a relationship between the generalized diffusion coefficient (${D}_{\nu }$) and the standard diffusion constant (*D*) given as

5 $${D}_{\nu }=\frac{D}{{\tau }_{D}^{\nu -1}}\,.$$

Equations (4) and (5), which relate the phage minimum sticking and diffusion times to the generalized and standard diffusion constants, were validated using a generic 2-dimensional CTRW scenario: $\dot{{\boldsymbol{x}}}(t)=\sqrt{2D}{\boldsymbol{W}}(t)$. For the simulations, $D=3.66\,\mu {{\rm{m}}}^{2}{\sec }^{-1}$ was the diffusion constant which was representative of a small T4 phage in water, with radius $R=60\,{\rm{nm}}$, at $25{{\rm{C}}}^{\circ }$, ${\tau }_{D}=0.05{\rm{s}}$ was the diffusion time (time step) and sticking times were drawn from the Pareto distribution $\psi (t;\nu ,{\tau }_{0})$ with ${\tau }_{0}$ related to $\nu$ using Eq. (4). To generate displacement times, $\psi (t;\nu ,{\tau }_{0})$ is first integrated from the minimum sticking time to the desired displacement time $\tau$ to get the cumulative distribution function ${\rm{cdf}}(\tau )={\int }_{{\tau }_{0}}^{\tau }\psi (t)\,dt=1-{({\tau }_{0}/\tau )}^{\nu }$.

The differential equation for $\dot{{\boldsymbol{x}}}(t)$ was numerically solved and subdiffusion induced by using a simple finite difference algorithm and a single scheduling process. In terms of the finite differences, $\dot{{\boldsymbol{x}}}$ was discretized by setting $\dot{{\boldsymbol{x}}}(t)=({{\boldsymbol{x}}}_{k+1}-{{\boldsymbol{x}}}_{k})/{\tau }_{D}$ and ${{\boldsymbol{x}}}_{k+1}$ solved for to get ${{\boldsymbol{x}}}_{k+1}={{\boldsymbol{x}}}_{k}+\sqrt{2D{\tau }_{D}}{\xi }_{k},$ where $k$ was the time step ($t=k{\tau }_{D}$) and the noise was discretely approximated^55^ as ${\boldsymbol{W}}(t)={\xi }_{k}/\sqrt{{\tau }_{D}}$ using Gaussian random numbers, ${\xi }_{k}$, of mean zero and unit variance. At $t=0$ the phage particle was initialized at the origin (${{\boldsymbol{x}}}_{0}=0$). Setting the equation for ${\rm{cdf}}(\tau )$ to a uniform random variable ${\rm{U}} \sim {\rm{Uni}}(0,1)$ between 0 and 1 and solving for $\tau$ gave the random displacement time $\tau ={\tau }_{0}{(1/{\rm{U}})}^{1/\nu }$. If $t < \tau$ the noise was held fixed at zero (${\xi }_{k}=0$) so the phage would remain stationary. Once $t\ge \tau$ a random Guassian number would be drawn and the phage would displace for a single time step, another displacement time would be drawn and the process would repeat. To produce the results, simulations would progress for 10^4^ time steps and 10^4^ replicate trajectories would be simulated.

From the simulated trajectories, the ensemble MSD $\langle {x}_{k}^{2}\rangle =\overline{{({x}_{i+k}-{x}_{i})}^{2}}$ was calculated and a log-log linear regression model $\log \,\langle {x}_{k}^{2}\rangle ={\beta }_{0}+{\beta }_{1}\,\log (\tau )+\varepsilon$, was applied to extract the trajectories effective diffusion coefficient (${K}_{\alpha }=\exp \,{\beta }_{0}$) and anomalous diffusion exponent $\alpha ={\beta }_{1}$^56^. The term $\varepsilon$ is a random variable accounting for unexplained random variation in the response $\log \,\langle {x}_{k}^{2}\rangle$. Figure 2A shows that as $\nu$ was varied between 0 to 2 then $\langle {x}_{k}^{2}\rangle  \sim {K}_{\alpha }\,{t}^{\alpha }$ where ${K}_{\alpha }=4D/{\tau }_{D}^{\alpha -1}$ as predicted by Eq. (5). Plotting the anomalous exponent *α* against the Pareto exponent $\nu$ (Fig. 2B) revealed $\alpha  \sim \nu$ in the region $0 < \nu  < 1$ (subdiffusion) and converged to one $(\alpha =1)$ for $\nu  > 1$ (diffusion). Thus, the simulated MSD asymptotics showed the diffusion exponent of the trajectories converged to the exponent in the Pareto distribution used to generate the displacement times.

### Phage and bacteria model calibration

The empirical values of the generalized diffusion constant $({D}_{\alpha })$ and anomalous diffusion exponent $(\alpha )$ for T4 wt and the standard diffusion constant (*D*) for T4Δ*hoc* (Table 1) were combined in Eq’s (4) and (5) with $\nu =\alpha$ and $d=3$ to estimate the typical diffusion time ${\tau }_{D}={(D/{D}_{\alpha })}^{1/\alpha -1}$ and minimum sticking time ${\tau }_{0}={\tau }_{D}\,sinc{(\alpha \pi )}^{1/\alpha }$ for different mucin concentrations (Fig. 2D). The diffusion time was similar across mucin concentrations with a typical value of ${\tau }_{D}\approx 0.01$ sec so that ${\tau }_{{\rm{relax}}}\ll {\tau }_{D}$. The minimum sticking time was also similar across mucin concentrations with a mean value of ${\tau }_{0}\approx 0.001$ sec.

Since ${\tau }_{{\rm{relax}}}\ll {\tau }_{D}$, the inertial term in Eq. (1) was neglected and the Stokes-Einstein relation applied to find

6 $${{\boldsymbol{v}}}_{P}(t)=\sqrt{2D}{\boldsymbol{W}}(t),$$

where *D* was set to the empirical values for T4Δ*hoc* listed in Table 1. Using *α* as the index in the Pareto distribution $\psi (\tau ;\alpha ,{\tau }_{0})$ would generate random walks of phage with ${\rm{MSD}}(t)=6{D}_{\alpha }{t}^{\alpha }$. Simulations of Eq. (6) recovered the expected transport properties measured experimentally at different mucin concentrations (Fig. 2E,f).

Similarly, since ${\tau }_{{\rm{relax}}}\ll {\tau }_{D}$ the inertial term in Eq. (2) was neglected. The thermal contribution of mucus to the bacteria’s velocity was close to that experienced in water and was orders of magnitude smaller than its typical propelling velocity, which yields

7 $${{\boldsymbol{v}}}_{B}(t)=\frac{1}{{\gamma }_{B}}{{\boldsymbol{f}}}_{B}(t).$$

This was the equation of motion used for the bacteria’s run regime for the propulsive term and run times (see Methods: bacteria motion in mucus).

### Encounter rate simulations: the impact of subdiffusion

Phage T4 and *E. coli* microbes were simulated by numerically integrating Eq’s (6) and (7) and applying the methods for bacteria-phage encounter rates as described in the methods section. The values of the physical and modeling parameters are listed in Table 5. The simulations were conducted for 1% (w/v) mucin, which was the reported maximum onset of T4 wt subdiffusion^32^. For all simulations, the analytical expressions indicated these results would be robust across mucus concentrations.

**Table 5:** Definitions of the physical and modeling parameters used in simulations of *E. coli* and T4 phage in mucus.

Parameter	Description	Value	Ref.
*R*_*P*_	T4 radius	0.09 *μ*m	Methods
*R*_*B*_	*E. coli* radius	1 *μ*m	^47^
*v*_*B*_	*E. coli* velocity	30 *μ*m/sec	^47^
*ω*_*T*_	*E. coli* tumble rate	1–100 sec^−1^	^47^
*τ*_*D*_	T4 diffusion time	0.01 sec	Methods
*V*	Simulation volume	10^6^ *μ*m^3^	Methods

#### Case 1:

**Figure 3:**
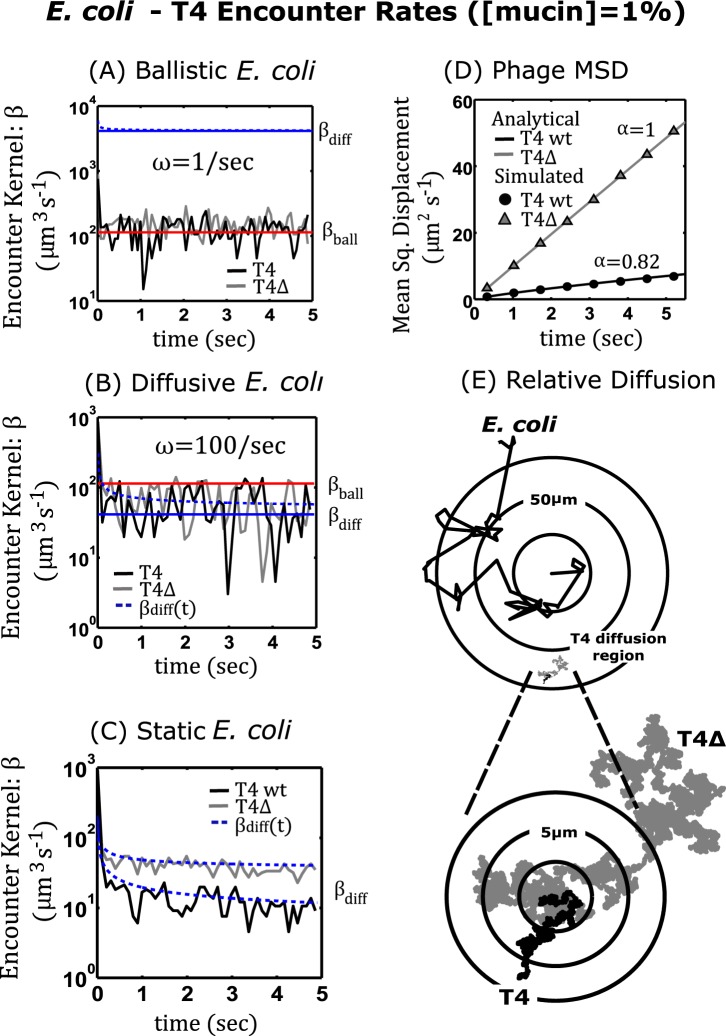
*E. coli*-T4 Encounter Rates $([{\rm{mucin}}]=1 \% )$. Panels (**A**–**C**) are plots of the *E. coli* -T4 encounters per unit time and the corresponding encounter kernels: *β*_ball_ (ballistic)–shown in red, *β*_diff_ (diffusive)–shown in blue, as a function of time (solid lines–steady state, dashed lines–transient) for three different motility regimes of *E. coli*. (**A**) The simulated data for ballistic *E. coli* with a small tumble frequency which physiologically corresponds to the bacterium swimming for long duration. (**B**) Diffusive *E. coli* with large tumble frequency or short swim times. (**C**) Static *E. coli* not swimming $({v}_{B}=0)$. The black curve corresponds to T4 wild type, and the grey curve corresponds to T4Δ*hoc*. (**D**) Analytical and simulated mean squared displacement for T4 wild type and T4Δhoc using the subdiffusion exponents observed empirically at $[{\rm{mucin}}]=1 \%$. (**E**) The bottom right image displays 2D projections of 43.3 sec trajectories for *E. coli*, T4 wild type, and T4Δ*hoc* at a ∼50 *μ*m scale. The inset displays both phage trajectories at a higher resolution (∼5 *μ*m).

***E. coli*** **Running and Tumbling**. When the *E. coli* bacterium operated within its typical run-and-tumble regime^47^ (${\omega }_{T}=1\,{\sec }^{-1}$), the simulated encounter rate kernels for T4 wt and T4Δ*hoc* appeared to vary around an average steady state over time (Fig. 3A). To interpret this result, it was noted that for time scales on the order of the *E. coli*’s average run time $\langle {\tau }_{R}\rangle$, its path is linear and the bacterium would travel a distance $\langle l\rangle ={v}_{B}\langle {\tau }_{R}\rangle =30\,\mu {\rm{m}}$. On the other hand, T4 has a root mean square (RMS) value of ${\rm{RMS}}=\sqrt{6{D}_{\alpha }{\tau }_{R}^{\alpha }}=2.49\mu {\rm{m}}$ (T4 wt) and $3.90\,\mu {\rm{m}}$ (T4Δ*hoc*) which are both an order of magnitude less than $\langle l\rangle$ (net distance traveled) and increases at a slower rate for $t\gg {\tau }_{R}$ since more time would be spent lingering or backtracking over areas already covered. Hence, the *E. coli* bacterium traveled a more ballistic path, whereas the T4’s transport was more diffusive. Put more explicitly, $\langle l\rangle \gg {\rm{RMS}}$, so the bacterium explored much more space than the phage (Fig. 3E). Thus, it was natural to suspect the encounter rates to be ballistic and primarily governed by the bacterium’s velocity and run time parameters. This interpretation motivated the calculation of a theoretical ballistic encounter kernel ${\beta }_{{\rm{ball}}}=\pi \,{R}_{C}^{2}\,{v}_{B}$. Computing *β*_ball_ with ${v}_{B}=30\,\mu {{\rm{msec}}}^{-1}$ and ${R}_{C}=1.1\,\mu {\rm{m}}$ gives ${\beta }_{{\rm{ball}}}=4.1\times {10}^{-7}$ (mLh^−1^) which agreed well with the simulation results (Fig. 3A solid red line).

#### Case 2:

***E. coli*** **with Short Run Times**. Next, the effect of phage subdiffusion on encounter rates when the bacterium’s motility was less dominate $(\langle l\rangle \ll {\rm{RMS}})$ was studied. For the simulations, the *E. coli* bacterium’s average run time was reduced by increasing the tumble frequency (${\omega }_{{\rm{T}}}=100\,{\sec }^{-1}$) in $\Phi ({\tau }_{R};{\omega }_{{\rm{T}}})$ resulting in a theoretical diffusion constant of ${D}_{B}={v}_{B}^{2}\langle {\tau }_{R}\rangle /3=3\times {10}^{-12}({{\rm{m}}}^{2}{\sec }^{-1})$ which was comparable to the empirical T4Δ*hoc* diffusion constant, $D=2.54\times {10}^{-12}({{\rm{m}}}^{2}{\text{sec}}^{-1})$ in 1% mucus. In this case, the simulated encounter rate kernels for both T4 wt and T4Δ*hoc* appeared to be bounded and convergent to average steady states that were on the same order of magnitude (Fig. 3B).

The simulation outputs were compared to the theoretical encounter rate approximation described in the methods section for two motile bacteria and phage species, given as

8 $${\mathscr{J}}(t)=4\pi {P}_{\infty }{R}_{C}\,{\mathfrak{D}}(t)(1+\sqrt{\frac{{R}_{C}^{2}}{\pi \,\lambda (t)}}),$$

where ${\mathfrak{D}}(t)={v}_{B}^{2}\langle {\tau }_{R}\rangle /3+\alpha {D}_{\alpha }{t}^{\alpha -1}$ and $\lambda (t)=\int {\mathfrak{D}}(t)\,dt$. Dividing Eq. (8) by ${P}_{\infty }$ gave the time dependent encounter rate kernel $\beta (t)$ which varies with both ${\mathfrak{D}}(t)$ and $\lambda {(t)}^{1/2}$. In the case of the T4 wt in 1% mucus; $\alpha =0.82$, so as $t$ gets large, ${\mathfrak{D}}\approx {v}_{B}^{2}\langle {\tau }_{R}\rangle /3$ and the terms in parentheses converge to one which indicated the bacterium’s diffusion primarily determined encounter rates over phage subdiffusion. The approximate encounter kernel for T4 wt computed with ${v}_{B}=30\,\mu {{\rm{msec}}}^{-1}$ and $\langle {\tau }_{R}\rangle =1/100\,\sec$ was ${\beta }_{{\rm{diff}}}=1.49\times {10}^{-7}$ (mLh^−1^). On the other hand, for the T4Δ*hoc*, $\alpha =1$ gave ${\mathfrak{D}}={v}_{B}^{2}\langle {\tau }_{R}\rangle /3+D$. Using $D=2.54\times {10}^{-12}({{\rm{m}}}^{2}{\text{sec}}^{-1})$ gave a T4Δ*hoc* encounter kernel of ${\beta }_{{\rm{diff}}}=2.77\times {10}^{-7}$ (mLh^−1^). Thus, when the bacterium tumbled frequently, the encounter rate kernels for both T4 wt and T4Δ*hoc* converged to average steady states (Fig. 3B solid blue line) that were close in magnitude and accurately predicted the simulation results.

#### **Case 3:**

***E. coli*** **Static**. For the static regime $({{\boldsymbol{f}}}_{B}=0)$, the analytical expression in Eq. (8) for the encounter kernel predicted that for a static bacterium the subdiffusive T4 wt would underperform against its diffusive counterpart T4Δ*hoc*, a result that was validated by the simulations (Fig. 3C). Here ${v}_{B}=0$ so ${\beta }_{{\rm{diff}}}=4\pi \,{R}_{C}(\alpha {D}_{\alpha }{t}^{\alpha -1})$. Setting $\alpha =0.82$ in Eq. (8) gave a decaying transient solution which is shown in Fig. 3C (blue dashed line). When $\alpha =1$ then the encounter rate kernel also had a transient solution (Fig. 3C blue dashed line) but stabilized to a value of ${\beta }_{{\rm{diff}}}=4\pi \,{R}_{C}D=1.26\times {10}^{-7}$ (mLh^−1^). Hence, the bacterium had a greater intake of T4Δ*hoc* (diffusion) over that of T4 wt (subdiffusion) even when it was static.

For the experiments in 1% mucin concentration and using a 10-minute adsoprtion assay with chips^32^, Barr *et al*. estimated the adsorption of T4 wt phage by *E. coli* in the laboratory as ${k}_{{\rm{T}}4}=2.8\times {10}^{-7}$ (mLh^−1^) and the control was $k=1.4\times {10}^{-7}$ (mLh^−1^). Both values of phage adsorption observed in the laboratory were on the same order of the values obtained from the simulations conducted in cases 1–3 above. This shows while the CTRW framework provides a mechanistic description of the phage-bacteria-mucus dynamics at the qualitative level, subdiffusion on its own can not explain the increased absorbtion of T4 wt by *E. coli*.

## Discussion

Subdiffusion has previously been correlated with an increase in bacteria-phage encounter rates in mucosal environments^32^. However, during the current study a most interesting result in relation to the BAM^32^ model’s argument for a subdiffusion mediated bacteria-phage encounter rate mechanism was demonstrated: high bacteria motility $({\omega }_{T}=1)$ renders bacteria-phage encounter rates almost independent of phage transport. Moreover, even if T4 wt subdiffuse $(\alpha  < 1)$ around lowly motile, often tumbling $({\omega }_{T}\gg 1)$ or static bacteria, then regular diffusion $(\alpha =1)$ is predicted to be a superior search strategy for the T4 phage. Therefore, subdiffusion is deemed a highly unlikely candidate for the mechanism that enhances bacteria-phage encounter rates in mucus as currently proposed by the BAM model. The importance of these results, both independently and in relation to the BAM model, can be better understood in the context of previous work on encounter rates between swimming organisms and viruses and the ensuing adsorption process^51,57,58^.

In a study on the uptake of aquatic viruses by microorganisms^57^, Murray and Jackson used the Sherwood number (Sh) as a proxy to calculate the factor by which diffusive transport is increased due to local fluid motion. In particular, they demonstrated how a 1 *μ*m particle, such as a bacterium, swimming at 50 *μ*msec^−1^ as opposed to being static, should experience only a very small increase $({\rm{Sh}}\approx 1)$ in its uptake of viruses diffusing regularly in the environment. Building on their work, the present study included subdiffusion into the viral uptake model in addition to the regular diffusion used by Murray and Jackson^57^. However, our simulations and calculations predict a *E. coli* bacterium, with typical run and tumble dynamics $({v}_{B}=30,\,{\omega }_{T}=1)$, should experience a higher uptake ratio of ${\beta }_{{\rm{ball}}}/{\beta }_{{\rm{diff}}}=3.25$ between the ballistic (*β*_ball_) to static (*β*_diff_) encounter rate kernels, *regardless* of phage diffusivity (see results: cases 1 and 3). Additionally, it was shown that due to the *E. coli*’s run and tumble dynamics, when the bacterium tumbled often with the same run velocity $({v}_{B}=30)$ but higher tumble frequency $({\omega }_{T}\gg 1)$, the bacterium’s viral uptake was decreased by almost half (see results: cases 1 and 2). The findings for this study elaborate on Murray and Jackson’s prior work^57^ by providing a more detailed mechanistic account of how bacterial motility dominates phage transport in regards to their encounter rates in mucus. Equally important, these findings describe encounter rates as an emergent population phenomena manifesting from the complex interactions of the underlying species on a scale where macroscopic fluid models lose resolution.

Our results also show both *E. coli*’s motility and phage diffusivity is not enough to account for the increased adsorption of T4 wt over the T4Δ*hoc* phage that Barr *et al*. observed in the laboratory^32^. Therefore, we reason how this may be accounted for, first in an analytical framework supported by classical work, and second in a more fine scale descriptive setting uniquely proposed in this study.

First, in the classical work “Encounter efficiency of coliphage-bacterium interaction”, author Arthur L. Koch revived the art of mathematical biology using the classical Smoluchoswki diffusion limited coagulation equation and collision theory^51^. Koch’s findings relied heavily on the use of a so called “effective radius” in the stationary solution of the Smoluchowski equation to account for discrepancies between encounter rates and the adsorption of viruses by bacteria in the laboratory^51^. Building upon Koch’s work and that of others^52,53,58^, the study presented here included subdiffusion into the Smoluchowski equation (Eq. (3)). Since subdiffusion was found to be an insufficient mechanism to boost T4 wt encounter rates, it is therefore supposed the virus may somehow change the physics of adsorption by increasing the effective radius through another mechanism by which subdiffusion is symptomatic. This new mechanism is different from the alternative hypothesis proposed by Abedon^58^ that binding to mucus simply allows phage to “sit and wait” for diffusing bacteria.

**Figure 4:**
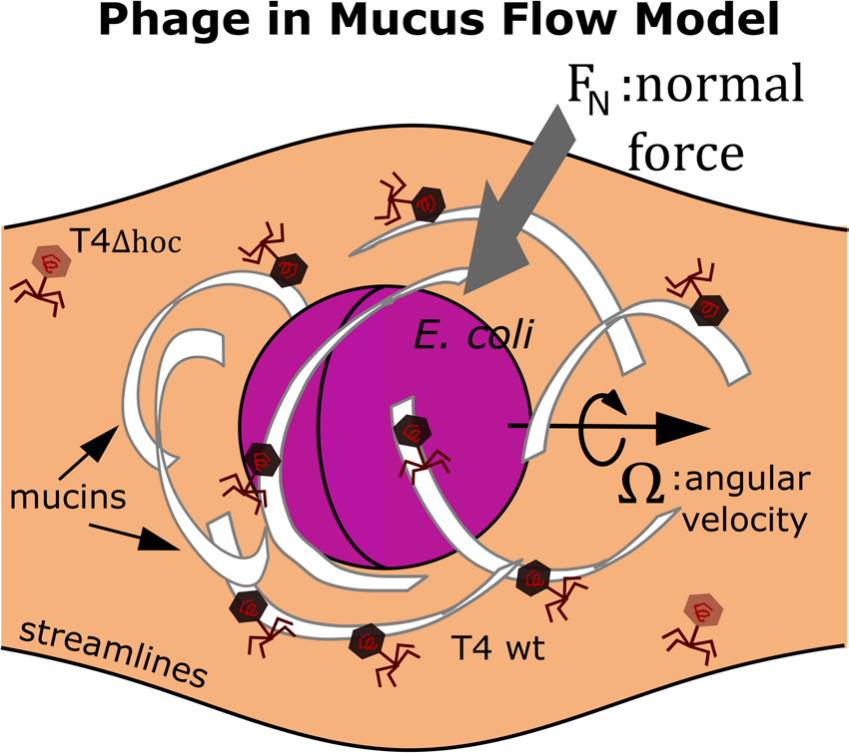
When the bacterium translates through the mucus medium in the direction of its angular velocity Ω, a normal force is generated, which pulls mucus polymers towards the bacterium.

Second, for this study, encounter rate simulations included only the geometry of encounters and the mechanism of diffusion for the rates. A more advanced formulation of the model could consider the fluid flow field around a swimming bacterium (see Fig. 4). Using the phage to effectively probe mucus showed the viscosity of low concentration mucus is similar to that of water (see: Methods), thus for concentrations <5% [mucin], mucus should behave similar to a Newtonian fluid^59^. It is hypothesized that the localized fluid flow around a bacterium with high motility $({\omega }_{T}=1)$ would generate near field drag forces^60^ that would cause the mucus polymer field to become deformed. The deformed polymer field should more efficiently pull T4 phages adhering with mucins toward the nearby bacterium. Since T4 phage has a ∼100 nm tail^31,44^ and provided the interaction with mucus might align T4 wt in a way through hydrophobic and electrostatic interactions that is more favorable for adsorption, the effective radius of adsorption for T4 wt may be greater than that for T4Δ*hoc*. This provides an alternative hypothesis in which *phage adherence to mucus* could work as a mechanism alongside *bacterial motility* to increase T4 wt adsorption in mucus. This hypothesis could be readily tested against static and swimming bacteria in the laboratory.

For this study, the CTRW modeling formalism proved to be a viable framework for simulating random walks with diffusion parameters as emergent phenomena. Using the calibration technology developed in this paper, cellular subdiffusion may also be modeled by CTRW using empirical data. CTRW shares many attractive features with diffusion in other biological structures such as bacteria in biofilms^61^ and tracers in crowded media such as living biological cells^62–64^. The analytical approximations used in this paper are generic, implying the essential observations for the encounter rates found herein should be similar for the case of other types of subdiffusive systems such as fractal Brownian motion.

## Supplementary information

Supplementary Information

## Data Availability

All materials, data and associated protocols contained in this manuscript will be made available in Github upon publication. Repository: https://github.com/luque82/Kevin_etal_SciRep_2019.

## Supplementary information

is available this paper at 10.1038/s41598-019-52794-2.
