# Supplementary material for: Impact of bacteria motility in the encounter rates with bacteriophage in mucus
Source: Sci Rep. 2019 Nov 11;9:16427. doi: 10.1038/s41598-019-52794-2 (PMC6848219; doi:10.1038/s41598-019-52794-2)
Supplement: Supplementary file 1 — Supplementary Information [file 41598_2019_52794_MOESM1_ESM.pdf]

# Supplementary Information

# Impact of bacteria motility in the encounter rates with bacteriophage in mucus

**Kevin L. Joiner[1,2,*], Arlette Baljon[3,7], Jeremy Barr[4], Forest Rohwer[5,7], and Antoni Luque[1,6,7,*]**

[1]Computational Science Research Center, San Diego State University, San Diego, CA 92182, USA

[2]Naval Information Warfare Center, San Diego, CA 92152, USA

[3]Department of Physics, San Diego State University, San Diego, CA 92182, USA

[4]School of Biological Sciences, Monash University, Clayton, VIC 3800, Australia

[5]Department of Biology, San Diego State University, San Diego, CA 92182, USA

[6]Department of Mathematics and Statistics, San Diego State University, San Diego, CA 92182, USA

[7]Viral Information Institute, San Diego State University, San Diego, CA 92182, USA

[*]Corresponding authors: kevin.l.joiner1@navy.mil, aluque@sdsu.edu

## Supplementary *E. coli* run-and-tumble computational model

Based on Netwon's laws of motion, the acceleration of the bacteria is determined by

$$m_B\, \boldsymbol{a}_B(t) = \boldsymbol{f}_B + \boldsymbol{f}_{\text{friction}} + \boldsymbol{f}_{\text{thermal}}.$$

Here, $m_B$ is the mass of the bacterium, that is, its resistance of being accelerated. The propelling force generated by the rotation of the flagella is $\boldsymbol{f}_B$; the friction term emerges from the fluid viscosity opposing the movement of the bacterium and can be approximated by $\boldsymbol{f}_{\text{friction}} \approx -\gamma_B \boldsymbol{v}_B(t)$, where $\gamma_B$ is the coefficient of friction for the bacterium, and $\boldsymbol{v}_B(t)$ is its velocity. Finally, the thermal force, $\boldsymbol{f}_{\text{thermal}}$, originates from the constant buffeting of fluid molecules due to their kinetic energy, which is proportional to the temperature, $T$; the average contribution of this stochastic term is zero, $\langle \boldsymbol{f}_{\text{thermal}} \rangle = 0$, but at each given time it provides on average a instantaneous kick $\langle f^i_{\text{thermal}}(t) f^j_{\text{thermal}}(t') \rangle = 2\ \gamma_B\ kT\ \ \delta_{i,j}\ \delta(t-t')$, where $i$ and $j$ can be 1 (x-axis), 2 (y-axis), and 3 (z-axis) depending on each component of the force, and 2 $\gamma_B$ $kT$ is the strength of the thermal force given by the fluctuation-dissipation theorem; $k$ is the Boltzmann constant. Thus, the velocity of the bacteria in the run regime is governed

by the Langevin equation:

$$\frac{d\boldsymbol{v}_B}{dt} = \frac{1}{m_B}\boldsymbol{f_B} - \frac{\gamma_B}{m_B}\boldsymbol{v}_B + \frac{\sqrt{2\gamma_B kT}}{m_B}\boldsymbol{\xi}(t), \tag{S.1}$$

where $\langle\boldsymbol{\xi}(\boldsymbol{t})\rangle = 0$ and $\langle\xi_i(t)\xi_j(t')\rangle = \delta_{ij}\delta(t-t')$. This first order stochastic equation is linear on $\boldsymbol{v}_B$, and $\tau_{relax} = m_B/\gamma_B$ represents the relaxation time in the exponential decay of the velocity when perturbed out of equilibrium (homogeneous equation). To estimate this relaxation time, one can use Stokes relation for the coefficient of friction $\gamma_B = 6\pi R_B \nu$. Taking the viscosity of water $\nu \approx 10^{-3}$ Pa sec, the mass of *E. coli* $m_B \approx 1$ pg, and approximating *E. coli* as a sphere of radius $R_B \approx 1$ $\mu$m, this leads to a relaxation time of $\tau_{relax} \approx 0.05$ $\mu$sec. This is four orders of magnitude smaller than the typical run time for *E. coli*, $\tau_R \sim 1$ sec. This suggests that the inertial term is not relevant, as far as the perturbation in the velocity is not drastic.

To estimate perturbation in the velocity the typical velocity change, $\Delta v_B$, induced by the kinetic energy of the fluid on the bacteria, one can assume a fluid similar to water, which is common in biology. The density of water is $\rho_{H_2O} \approx 1$ kg dm$^{-3}$. The mass of a water molecule is $m_{H_2O} = 18$ g mol$^{-1}$. Thus, the typical distance between fluid molecules is $l \approx 3\cdot 10^{-10}$ m. In thermal equilibrium, each water molecule have a typical velocity $v_{H_2O} \approx \sqrt{kT/m_{H_2O}} \approx 371$ m sec$^{-1}$ (standard temperature $T = 273.15$ K. This defines the collision time scale $\tau_{col} \sim l/v_{H_2O} \approx 8.37\cdot 10^{-13}$ sec. From the Langevin equation above, the contribution of the thermal bath into the velocity is $\Delta v \approx \sqrt{2\gamma_B kT\tau_{col}}/m_B \approx 10^{-2}$ $\mu$m sec$^{-1}$.

The typical velocity generated by the propelling mechanism of the bacteria is $v_B \sim 10$ $\mu$m/s, which is three orders of larger than the thermal perturbation. Since the relaxation time and change in velocity is orders of magnitude smaller than the typical run time and propelling velocity of the bacterium, one can neglect the inertial term $d\boldsymbol{v}/dt$ and the thermal term in Eq. (S.1). This reduces the equation of motion for the run regime to $\boldsymbol{v}_B \approx \boldsymbol{f_B}/\gamma_B = \boldsymbol{v}_{emp}$ where $\boldsymbol{v}_{emp}$ is bacterial speed measured empirically, which acts in the longitudinal direction of the bacterium. Thus, the position of the bacteria is determined integrating the equation

$$\frac{d\boldsymbol{x}_B}{dt} = \boldsymbol{v}_{emp}.$$

Then, the final position of a bacterium after each run is $\boldsymbol{x}_B(t+\tau_R) = \boldsymbol{v}_{emp}\,\tau_R + \boldsymbol{x}_B(0)$, where $\tau_R$ is distributed exponentially with a mean time $\langle\tau_R\rangle$. This is determined by the probability distribution function $\Phi(\tau_R) = \omega_R\, e^{-\omega_R\tau_R}$, where the mean run rate is $\omega_R = 1/\langle\tau_R\rangle$. The term $\vec{x}_B(0)$ is the position of the bacteria at the beginning of the run.

In the tumble regime, *E. coli* stops swimming and rotates the flagella in the opposite direction, disassembling the bundle and moving erratically. The mean tumble time is about $\tau_T \sim 0.1$ sec, which is an order of magnitude shorter than the running time. The longitudinal axis of *E. coli* reorients on average $\langle\theta_T\rangle \approx 70°$. While tumbling, the bacteria can diffuse due to the thermal fluctuations of the fluid. Using Einstein's relation from the dissipation-fluctuation theorem one can estimate the diffusion of the non-swimming bacteria, $D_B = kT/\gamma \approx 0.22$ $\mu$m$^2$ m sec$^{-1}$. The mean-squared displacement of the bacteria during the tumbling period is proportional to the bacterial diffusion and the tumbling time, $MSD = 6D_B\tau_T \approx 0.13$ $\mu$m$^2$ sec$^{-1}$. The typical

displacement of the bacteria during the tumbling period is $\Delta r \sim \sqrt{MSD} \approx 0.36$ $\mu$m. This is an order of magnitude smaller than the actual size of *E. coli*. Thus, the displacement during the tumbling regime is negligible, and it is not included in the model. For the sake of computation efficiently, instead of using a Langevin equation of motion for the rotation, the tumbling process is generated probabilistically. The mean tumbling time is an order of magnitude shorter than the run time. In a first approximation, the tumbling regime is evaluated instantaneously. The change of direction is approximated from a uniform distribution between 0 and $2\pi$,

$$U(\theta_T) = \frac{1}{2\pi} .$$

Thus, in the computational model, the updated directional change of the bacterium after a tumble event is $\theta(t+\delta t_T) = \theta(t) + \theta_T$, where $\delta t_T \to 0$ indicates that the step is virtually instantaneous.

## Supplementary Asymptotic Mean Squared Displacement for Continuous Time Random Walks

Based on experimental data, it is plausible to assume that the mechanism underlying phage subdiffusion in mucus emerges from the interaction of phage with mucus fibers and the subsequent diffusive excursion until encountering a new fiber. This mechanistic model can be studied using probabilistic theory to obtain a theoretical expression of the phage subdiffusion that facilitates the interpretation of experiments and the calibration of computational models.

The time of one production step is $\tau = \tau_W + \tau_D$, where $\tau_W$ is the waiting time spent by the phage adhering to a mucus fiber, and $\tau_D$ is the time spent diffusing between the release from the mucus fiber and binding to another fiber. In this context, the probability of finding a phage between $x$ and $x + dx$ at time $t$ is

$$P(x,t)dt = \sum_{i=1}^{\infty} P(t;n)P_n(x)dt. \tag{S.2}$$

which is the basic CTRW transport equation. Here, $t = \sum_{i=1}^{n} \tau_i$ is the time after $n$ production steps, $P(t;n)$ is the probability of producing $t$ in $n$ steps, and $P_n(x)$ is the probability density function (*pdf*) of being at $x$ after $n$ diffusive steps. To evaluate the probability of finding a phage at $x$ at time $t$, one needs to consider all possible trajectories, that is, any possible number of steps $n$, which leads to the sum in Eq. (S.2). The goal is to estimate the mean square displacement, which will require to estimate the first and second moments of $x$, $\langle x(t) \rangle = \int_{-\infty}^{\infty} xP(x,t)dt$ and $\langle x^2(t) \rangle = \int_{-\infty}^{\infty} x^2 P(x,t)dt$. As shown below, it is possible to obtain an analytic expression for the MSD in the asymptotic limit, that is, for $t$ large.

Renewal theory allows to express the *pdf* $P_n(x)$ and the probability $P(t;n)$ in terms of underlying physical processes. The total displacement, $x$, is the sum of $n$ displacements, $\Delta x$ so that $x = \sum_{i=1}^{n} \Delta x_i$. If the displacements $\Delta x_i$ are independent,

identically distributed processes (*i.i.d.*) generated by the *pdf* $f(\Delta x)$, then $P_n(x)$ is given by

$$P_n(x) = \int_{-\infty}^{\infty} d\Delta x_1 f(\Delta x_1) \int_{-\infty}^{\infty} d\Delta x_2 f(\Delta x_2) \cdots \int_{-\infty}^{\infty} \Delta x_{n-1} f(\Delta x_{n-1}) f(\Delta x_n(x)) \ ,$$

where $\Delta x_n(x) = x - \sum_{i=1}^{n-1} \Delta x_i$. Applying the convolution theorem for Fourier transforms gives $\tilde{P}_n(k) = \left(\tilde{f}(k)\right)^n$, where $\tilde{f}(k) = \int_{-\infty}^{\infty} e^{ik\Delta x} f(\Delta x) d\Delta x$ is the Fourier transform of $f(\Delta x)$. This result shows in Fourier space the *pdf* of the total displacement is just the product of the underlying *pdf*s for individual displacements. Thus, the goal *pdf* $P(x,t)$ in Eq. (S.2) can be expressed in Fourier space as

$$\tilde{P}(k,t) = \sum_{i=0}^{\infty} P(t;n) \left(\tilde{f}(k)\right)^n \ .$$

Recall that $P(t;n)$ is the probability of having $n$ events within $(0,t)$, that is, $0 < t_1 < t_2 < \cdots < t_n < t < t_{n+1}$, where the waiting time is defined by consecutive events $\tau_i = t_i - t_{i-1}$. The sequence of waiting times, $\{\tau_i\}$, are *i.i.d.* random variables with *pdf* $\psi(\tau)$, which is called the renewal process. The probability $P(t;n)$ can be expressed as an indicator function $P(t;n) = \langle I(t_n < t < t_{n+1}) \rangle$ where $I = 1$ if $t_n < t < t_{n+1}$ and $I = 0$ otherwise. The stochastic variable $t$ is larger than zero, so it is convenient to use the Laplace transform to achieve

$$\hat{P}(s;n) = \int_0^{\infty} \langle I(t_n < t < t_{n+1}) \rangle e^{-st} dt = \langle \int_{t_n}^{t_{n+1}} e^{-st} dt \rangle = \frac{\langle e^{-st_n} \rangle - \langle e^{-st_{n+1}} \rangle}{s} \ . \tag{S.3}$$

In the first step of the above result, the linear properties of the average ensemble operator, $\langle \cdot \rangle$ is applied and the fact that the indicator function is one within $t_n$ and $t_{n+1}$ but zero elsewhere. Noticing that $t_n = \sum_{i=1}^{n} \tau_i$ for the *i.i.d.* random variables $\tau_i$, the average terms on the right hand side can be expressed in terms of the Laplace transform of the renewal process: $\hat{\psi}(s) = \int_0^{\infty} \psi(\tau) e^{-s\tau} d\tau$ as

$$\langle e^{-st_n} \rangle = \langle e^{-s\sum_{i=1}^{n} \tau_i} \rangle = \prod_{i=1}^{n} \langle e^{-s\tau_i} \rangle = \prod_{i=1}^{n} \langle \int_0^{\infty} \psi(\tau_i) e^{-s\tau} d\tau_i \rangle = (\hat{\psi}(s))^n \ ,$$

where the same applies for the term $t_{n+1}$ so that $\langle e^{-st_{n+1}} \rangle = (\hat{\psi}(s))^{n+1}$. Applying this result in Eq. (S.3), gives

$$\hat{P}(s,n) = \frac{1 - \hat{\psi}(s)}{s} (\hat{\psi}(s))^n \ . \tag{S.4}$$

Now, it is possible to express the goal *pdf*, $P(x,t)$, in Eq. (S.2), in terms of the underlying mechanistic processes. Applying the Fourier and Laplace transforms to $P(x,t)$ leads to

$$\hat{\tilde{P}}(k,s) = \sum_{n=0}^{\infty} \frac{1 - \hat{\psi}(s)}{s} (\hat{\psi}(s))^n \left(\tilde{f}(k)\right)^n = \frac{1 - \hat{\psi}(s)}{s} \sum_{n=0}^{\infty} \left(\hat{\psi}(s)\tilde{f}(k)\right)^n \ ,$$

which is a geometric series that converges under the reasonable assumption $|\hat{\psi}(s)\tilde{f}(k)| < 1$. The initial *pdf*s, $\psi(t)$ and $f(\Delta x)$ are normalized, which implies $|\hat{\psi}(s)| \leq 1$ and $|\tilde{f}(k)| \leq 1$, where the only cases have the equality would be for delta functions. Thus, except for single delta distributions the geometric series above will converge, leading to

$$\hat{\tilde{P}}(k,s) = \frac{1-\hat{\psi}(s)}{s\left(1-\hat{\psi}(s)\tilde{f}(k)\right)} \ .$$

which can be used to calculate the moments for the total displacement, which is the main goal of this derivation.

The expansion of the exponential kernel of the Fourier transform allows to express a *pdf* in terms of the moments of the variable. The expression is given by

$$\hat{\tilde{P}}(k,s) = \sum_{p=0}^{\infty} \frac{i^p}{p!} \langle \hat{x}^p(s) \rangle k^p \ . \tag{S.5}$$

This expression also allows to express the moments in terms of the derivatives of the transformed *pdf*: $\langle \hat{x}^p(s)\rangle = (-i)^p \, \partial^p \hat{\tilde{P}}(k,s)/\partial k^p|_{k=0}$. The particular expressions for the first and second moments are, respectively,

$$\langle \hat{x}(s) \rangle = -i \frac{\partial \hat{\tilde{P}}(k,s)}{\partial k}\bigg|_{k=0} \quad \text{and} \quad \langle \hat{x^2}(s) \rangle = -\frac{\partial^2 \hat{\tilde{P}}(k,s)}{\partial k^2}\bigg|_{k=0} . \tag{S.6}$$

Before diving in the calculation of these moments, it is important to emphasize the following properties of $\tilde{f}(k)$, which emerge from being the Fourier transform of the *pdf* $f(\Delta x)$):

$$\frac{d\tilde{f}(k)}{dk} = i\langle e^{ik\Delta x} \rangle \quad \text{and} \quad \tilde{f}(0) = 1 \ .$$

Now, applying the expression of $\hat{\tilde{P}}(k,s)$ from Eq. (S.5) in the equations for the first moment $\langle \hat{x}(s) \rangle$ and using the properties stated above, one obtains

$$\langle \hat{x}(s) \rangle = \frac{\hat{\psi}(s)}{1-\hat{\psi}(s)} \Delta x.$$

The prefactor related to the transformed renewal process, $\hat{\psi}(s)$, can be related to the average number of steps taken, $\langle \hat{n}(s) \rangle$. The average number of renewal steps within time $(0,t)$ is related to the probability $P(t;n)$ as $\langle n(t) \rangle = \sum_{n=0}^{\infty} nP(t;n)$ with Laplace transform:

$$\langle \hat{n}(s) \rangle = \sum_{n=0}^{\infty} n\hat{P}(s;n) = \frac{1-\hat{\psi}(s)}{s} \sum_{n=0}^{\infty} n \left(\hat{\psi}(s)\right)^n \ .$$

This is a known arithmetico-geometric sequence, $\sum_{n=0}^{\infty} nr^n = r/\left(1-r\right)^2$, where $r = |\hat{\psi}(s)|$ and convergences when $|\hat{\psi}(s)| < 1$,

which, as discussed above, is a common condition for most renewal processes. Then,

$$\langle \hat{n}(s) \rangle = \frac{\hat{\psi}(s)}{s\,(1-\hat{\psi}(s))} \ . \tag{S.7}$$

Going back to the expression for $\langle \hat{x}(s) \rangle$, this leads to $\langle \hat{x}(s) \rangle = \langle \hat{n}(s) \rangle \langle \Delta x \rangle$ which can be inverted using the Laplace transform to obtain the first moment of the total displacement

$$\langle x(t) \rangle = \langle n(t) \rangle \langle \Delta x \rangle,$$

which is a very intuitive result. On average, the total displacement is the average number of steps produced within $(0,t)$ times the average individual displacement. In the absence of drift, that is, $\langle \Delta x \rangle = 0$, the total average displacement is zero, $\langle x(0) \rangle = 0$. This is a relevant result for the research presented here.

Let's address now the second moment, which is necessary to determine the MSD and obtain a mechanistic expression for subdiffusion in the context of CTRW. By applying the expression of $\hat{\hat{P}}(k,s)$ given by Eq. (S.5) in the equation for $\langle \hat{x}(s) \rangle$, Eq. (S.6), one obtains

$$\langle \hat{x^2}(s) \rangle = \frac{\hat{\psi}(s)}{s\,(1-\hat{\psi}(s))} \langle \Delta x^2 \rangle + \frac{2(\hat{\psi}(s))^2}{s\,(1-\hat{\psi}(s))^2} \langle \Delta x \rangle^2$$

The prefactor of the first term of in the right hand side is $\langle \hat{n}(s) \rangle$, as derived above. The prefactor in the second term can be also related to averages of $\hat{n}(s)$. The following technique illustrates this and is also convenient when generating higher order moments of $\hat{x}(s)$. The key is to focus on the following generating function $h(r) = 1/1-r = 1+r+r^2 \cdots = \sum_{n=0}^{\infty} r^n$, which can be obtained by expanding $h(r)$ in a Taylor series around $r=0$. Taking the first derivative of $h(r)$ and multiplying both sides by $r$ gives $\sum_{n=0}^{\infty} n r^n = r/(1-r)^2$, which is the arithmetico-geometric series used to obtain the first moment of $\hat{n}(s)$ above. An analogous process with the second derivative of $h(r)$ leads to $\sum_{n=0}^{\infty} n(n-1) r^n = 2r^2/(1-r)^3$. Using these expressions in the equation for $\langle \hat{x}^2(s) \rangle$, one obtains

$$\langle \hat{x^2}(s) \rangle = \frac{1-\hat{\psi}(s)}{s} \frac{\hat{\psi}(s)}{(1-\hat{\psi}(s))^2} \langle \Delta x^2 \rangle + \frac{1-\hat{\psi}(s)}{s} \frac{2(\hat{\psi}(s))^2}{(1-\hat{\psi}(s))^3} \langle \Delta x \rangle^2,$$

which can be expressed in terms of series with the identities derived above

$$\langle \hat{x^2}(s) \rangle = \sum_{n=0}^{\infty} n(\hat{\psi}(s))^n \frac{1-\hat{\psi}(s)}{s} \langle \Delta x^2 \rangle + \sum_{n=0}^{\infty} n(n-1)(\hat{\psi}(s))^n \frac{1-\hat{\psi}(s)}{s} \langle \Delta x \rangle^2 \ .$$

Applying the Eq. (S.4) for $P(\hat{s};n)$, leads to

$$\langle \hat{x^2}(s) \rangle = \sum_{n=0}^{\infty} n\hat{P}(s;n) \langle \Delta x^2 \rangle + \sum_{n=0}^{\infty} n(n-1)\hat{P}(s;n) \langle \Delta x \rangle^2,$$

which gives the insightful expression for the second moment

$$\langle \hat{x^2}(s) \rangle = \langle \hat{n}(s) \rangle \langle \Delta x^2 \rangle + \langle \hat{n}(s)(\hat{n}(s)-1) \rangle \langle \Delta x \rangle^2.$$

The Laplace transform of this expression leads to

$$\langle x^2(t) \rangle = \langle \hat{n}(t) \rangle \langle \Delta x^2 \rangle + \langle \hat{n}(t)(\hat{n}(t)-1) \rangle \langle \Delta x \rangle^2,$$

which was the main goal of this calculation. Notice that this general result has a neat physical interpretation. The second moment of the displacement is determined by two contributions. The first term is related to the fluctuations of the local displacement $\Delta x$ and the second term to the fluctuations of the number of steps produced within $(0,t)$.

Now the only step missing is to assign the type of local displacement, that is, the *pdf* $f(\Delta x)$, as well as the renewal process, that is, the *pdf* $\psi(\tau)$. In the research problem addressed here, the excursion of phages are assumed to be diffusive and have no drift. Then

$$\langle \Delta x \rangle = 0 \quad \text{and} \quad \langle \Delta x^2 \rangle = 2D\tau_D,$$

where $D$ is the diffusion constant and $\tau_D$ is a set diffusive time, which, as shown later, it needs to be adjusted to reproduce the empirical subdiffusion of phages. Thus, the moments of $x(t)$ reduce to $\langle x(t) \rangle = 0$ and $\langle x^2(t) \rangle = 2D\tau_D \langle n(t) \rangle$. In this case, the MSD is

$$\text{MSD}(t) = \langle (x(t)-x(0))^2 \rangle = \langle x^2(t) \rangle = 2D\tau_D \langle n(t) \rangle \ . \tag{S.8}$$

The final task of the calculation reduces to estimate $\langle n(t) \rangle$. The goal is to obtain a mechanistic expression for the subdiffusion, that is,

$$\text{MSD}(t) = K_\alpha t^\alpha \ , \tag{S.9}$$

where one can relate the subdiffusion exponent $\alpha$ and the prefactor $K_\alpha$ with the underlying microscopic properties of the system. From the expression derived above for MSD$(t)$, the objective is to obtain an average number of steps within $(0,t)$ that is proportional to time to the $\alpha$, that is: $\langle n(t) \rangle \propto t^\alpha$. By reverse engineering, one can show that, in the asymptotic limit (large $t$), this result emerges from a renewal process following a power law distribution, that is,

$$\psi(\tau) \propto \frac{1}{\tau^{1+\alpha}} \ .$$

Taking the above as an ansatz it is possible to derive the expressions. The renewal process $\psi(\tau)$ is a *pdf* which must be normalized. The subdiffusion exponent is positive by construction, $\alpha > 0$. This forces to define a minimum time, $\tau_0$, to guarantee the normalization of $\psi(\tau)$ expressed as $\int_{\tau_0}^{\infty} \psi(\tau) d\tau = 1$. Then,

$$\psi(t) = \begin{cases} \frac{A}{\tau^{1+\alpha}} = \frac{\alpha}{\tau_0} \left(\frac{\tau_0}{\tau}\right)^{1+\alpha} & \tau > \tau_0 \\ 0 & 0 < \tau < \tau_0 \end{cases} ,$$

where $A = \alpha \tau_0^{\alpha}$. Mathematically it is more neat to take $\tau_0$ as the unit time. But physically, it is convenient to keep it explicit during the derivation to keep track of the units and interpret better the assumptions that will be involved. The average number of steps in the Laplace space, $\langle \hat{n}(s) \rangle$, depends only on the Laplace transform of the renewal process, Eq. (S.7). In this case, the Laplace transform of $\psi(t)$ is $\hat{\psi}(s) = \alpha \tau_0^{\alpha} \int_{\tau_0}^{\infty} \tau^{-1-\alpha} e^{-s\tau} d\tau$. If the moments of the renewal process were defined, one could use an analogous expansion of the exponential kernel to express $\hat{\psi}(s)$ as done for the Fourier transform case. Unfortunately, for the cases of interest here $0 < \alpha < 1$, all the moments diverge. Alternatively, it is possible to find an asymptotic form that follows $\hat{\psi}(s) \sim 1 + Cs^{\alpha} o(s)$ for small $s$ which is key to obtain the subdiffusion form of the MSD. To achieve this, it is convenient to express this integral in terms of gamma functions by applying the change of variables $u = s\tau$ and integrate by parts to get

$$\hat{\psi}(s) = \alpha (s\tau_0)^{\alpha} \int_{s\tau_0}^{\infty} u^{-1-\alpha} e^{-u} du = e^{-s\tau_0} - \alpha (s\tau_0)^{\alpha} \int_{s\tau_0}^{\infty} u^{-\alpha} e^{-u} du \ .$$

The integral in the right hand side of the second equality has the form of an upper incomplete gamma function $\hat{\psi}(s) = e^{-s\tau_0} - \alpha (s\tau_0)^{\alpha} \Gamma(1-\alpha, s\tau_0)$. The upper incomplete gamma function can be related to the gamma function and the lower incomplete gamma function $\Gamma(1-\alpha, s\tau_0) = \Gamma(1-\alpha) - \gamma(1-\alpha, s\tau_0)$. The lower incomplete gamma function can be developed in terms of holomorphic functions as

$$\gamma(1-\alpha, s\tau_0) = (s\tau_0)^{1-\alpha} \Gamma(1-\alpha) e^{-s\tau_0} \sum_{k=0}^{\infty} \frac{(s\tau_0)^k}{\Gamma(k-\alpha)} \ .$$

Applying this in the renewal process transform, one obtains

$$\hat{\psi}(s) = e^{-s\tau_0} - \alpha (s\tau_0)^{\alpha} \Gamma(1-\alpha) + (s\tau_0) \Gamma(1-\alpha) e^{-s\tau_0} \sum_{k=0}^{\infty} \frac{(s\tau_0)^k}{\Gamma(k-\alpha)} \ .$$

In the asymptotic limit for $s\tau_0$ small (large times), one can expand the exponential in a Taylor series and compare terms. The exponent is constrained, $0 < \alpha < 1$, so the smallest order of the expansion is $(s\tau_0)^{\alpha}$. Then the asymptotic expression for the renewal process is

$$\hat{\psi}(s) \sim 1 - \Gamma(1-\alpha)(s\tau_0)^{\alpha} + o(s\tau_0) \ .$$

Applying this result in Eq. (S.7), one obtains the asymptotic result

$$\langle \hat{n}(s) \rangle \sim \frac{1}{\tau_0^{\alpha}\Gamma(1-\alpha)s^{\alpha+1}} - \frac{1}{s} \ .$$

The inverse Laplace transform of $\langle \hat{n}(s) \rangle$ leads to

$$\langle n(t) \rangle \sim \frac{t^{\alpha}}{\tau_0^{\alpha}\Gamma(1-\alpha)\Gamma(1+\alpha)} - 1 \ .$$

Using the the reflection relation

$$\Gamma(1+\alpha)\Gamma(1-\alpha) = \frac{\alpha\pi}{\sin(\alpha\pi)} = \frac{1}{\mathrm{sinc}\,(\alpha)},$$

one obtains

$$\langle n(t) \rangle \sim \mathrm{sinc}\,(\alpha)\left(\frac{t}{\tau_0}\right)^{\alpha} - 1.$$

The unit term on the right hand of the first equation is relevant only when the power exponent $\alpha$ is infinitely small: $\lim_{\alpha\to 0}\langle n(t) \rangle \sim 0$. Thus, when the renewal process has an exponent that tends to one, the effective number of average steps is null. Notice that $\psi(t) = A/\tau$ can't be normalized because its integral is a logarithm and its upper limit does not decay to zero, that is, it is infinite. Physically, this translates into infinite waiting times, that is, no production steps.

For situations when $t \gg \tau_0$, it follows $(\tau_0/t)^{\alpha} \approx 0$ so that $\mathrm{sinc}\,(\alpha) \gg (\tau_0/t)^{\alpha}$. In this case,

$$\mathrm{sinc}\,(\alpha)\left(\frac{t}{\tau_0}\right)^{\alpha} - 1 = \left(\frac{t}{\tau_0}\right)^{\alpha}\left[\mathrm{sinc}\,(\alpha) - \left(\frac{\tau_0}{t}\right)^{\alpha}\right] \approx \mathrm{sinc}\,(\alpha)\left(\frac{t}{\tau_0}\right)^{\alpha} \ .$$

So that

$$\langle n(t) \rangle \sim \mathrm{sinc}\,(\alpha)\left(\frac{t}{\tau_0}\right)^{\alpha} ; \quad t \gg \tau_0 \tag{S.10}$$

Now, in the case for when $t = \tau_D$, by construction, the particle diffuses one step ($\langle n(t) \rangle = 1$) giving the relation

$$1 = \mathrm{sinc}\,(\alpha)\left(\frac{\tau_D}{\tau_0}\right)^{\alpha} ; \quad \tau_D \gg \tau_0 \tag{S.11}$$

This provides all the elements to obtain the mean squared displacement in the absence of drift, Eq. (S.8),

$$\mathrm{MSD}(t) \sim 2D\tau_D\left[\mathrm{sinc}\,(\alpha)\left(\frac{t}{\tau_0}\right)^{\alpha} - 1\right] \approx \begin{cases} 2D\tau_D\,\mathrm{sinc}\,(\alpha)\left(\frac{t}{\tau_0}\right)^{\alpha}; & \alpha > 0, \quad t \gg \tau_0 \\ 0 & \alpha \to 0 \end{cases} \ .$$

This result has been calculated for a single dimension. If the same process applies in multiple dimensions then, one must add a similar term for each dimension, for example, in 3D $\langle r^2(t)\rangle = \langle x^2(t)\rangle + \langle y^2(t)\rangle + \langle z^2(t)\rangle = 3\langle x^2(t)\rangle = d\langle x^2(t)\rangle$ with $d = 3$. Then,

$$\mathrm{MSD}(t) \sim 2dD\tau_D \left[\mathrm{sinc}\,(\alpha)\left(\frac{t}{\tau_0}\right)^{\alpha} - 1\right] \approx \begin{cases} 2dD\tau_D\,\mathrm{sinc}\,(\alpha)\left(\frac{t}{\tau_0}\right)^{\alpha}; & \alpha > 0, \quad t \gg \tau_0 \\ 0 & \alpha \to 0 \end{cases} .$$

This provides a mechanistic way to interpret the generalized diffusion coefficient, $K_\alpha$, in Eq. (S.9),

$$K_\alpha \approx 2dD\,\mathrm{sinc}\,(\alpha)\frac{\tau_D}{\tau_0^{\alpha}} \ . \tag{S.12}$$

Solving for $\tau_0$ in the above via Eq. (S.11) gives the remarkable formula

$$K_\alpha \approx \frac{2dD}{\tau_D^{\alpha-1}}; \quad \tau_D \gg \tau_0, \quad \alpha > 0 \tag{S.13}$$

In this expression, in the limit $\alpha \to 1$, the generalized diffusion recovers the regular diffusion prefactor in the MSD, that is, $\lim_{\alpha\to 1} D_\alpha = 2dD$. In this limit, the time corresponds to the number of steps diffusing, that is, $t \to n\tau_D$. Then,

$$\lim_{\alpha\to 1} \mathrm{MSD}(t) = \lim_{\alpha\to 1} 2dDn^{\alpha}\tau_D\,\mathrm{sinc}\,(\alpha\pi)\left(\frac{\tau_D}{\tau_0}\right)^{\alpha} = 2dDn\tau_D \ ,$$

This provides a very neat interpretation of the subdiffusion coefficient: It is just the regular diffusion coefficient divided by the diffusion time to the alpha. This provides also a mechanistic way to write the MSD subdiffusion equation:

$$\mathrm{MSD}(t) = \frac{2dD\tau_D}{\tau_D^{\alpha}}t^{\alpha} = 2dD\tau_D\left(\frac{t}{\tau_D}\right)^{\alpha} \ . \tag{S.14}$$

This offers a very insightful look at subdiffusion: It's just regular diffusion rescaled by a power function of $\alpha$ for time in units of the diffusion time. The dimension $(d)$ and diffusion $(D)$ can usually be obtained by independent experiments or first principles. Then, by fitting the MSD data to the equation above, one can obtain an empirical value for the diffusive time, $\tau_D$. This, in turn, is related to the minimum waiting time, $\tau_0$ by Eq. (S.11). Thus, Eq. (S.14) provides a direct way to extract the molecular times of a subdiffusive process assuming CTRW. These timescales can be combined with molecular models (binding, polymeric separation) to extract even more valuable information from (sub)diffusion data.

Let's now show that the results obtained above are self-consistent. In particular, for $\alpha \to 1$, it was claimed that $t \to n\tau_D$, that is, the majority of steps tend to be diffusive. One way to confirm this, is to calculate the probability of generating a waiting

time, $\tau_w$, smaller than the diffusion time, $\tau_D$:

$$P(\tau_w < \tau_D) = \int_{\tau_0}^{\tau_D} \psi(\tau)d\tau = 1 - \left(\frac{\tau_0}{\tau_D}\right)^{\alpha} = 1 - \mathrm{sinc}(\alpha) \ ,$$

where the identify in Eq. (S.11) has been used in the second equality. The probability of generating a waiting time is smooth monotonous function that grows from zero ($\alpha \to 0$) to one ($\alpha \to 1$). Thus, as the subdiffusive exponent approaches to one, the diffusive time will dominate, showing that the identify derived above for both time scales is self-consistent.

Another straightforward way to confirm self consistency is to use the identity in Eq. (S.11) to solve for $\tau_0$ in Eq. (S.10) to get

$$\langle n(t) \rangle \sim \frac{t^{\alpha}}{\tau_D^{\alpha}}; \quad t \gg \tau_0. \tag{S.15}$$

Letting $\alpha \to 1$ gives $t \sim \langle n(t) \rangle \, \tau_D$ which shows the identity derived above for both time scales is self-consistent.

## Supplementary Diffusive Encounter Rate Approximation of Two Motile Species

Here we develop a diffusive encounter rate model for phage-bacteria interactions based on their motility patterns. We assume the bacteria undergoes a random walk $\boldsymbol{w}_B(t)$ while the phage has a random walk $\boldsymbol{w}_P(t)$. As illustrated in panel (b), adopting a rest frame centered on the bacteria, we can form an equivalent random walk $\boldsymbol{w}(t) = \boldsymbol{w}_B(t) - \boldsymbol{w}_P(t)$. The square of the distance of the phage from the bacteria is $r^2 = (x_B - x_P)^2 + (y_B - y_P)^2 + (z_B - z_P)^2$. Without loss of generality, the mean of $r^2$ can be evaluated by first expanding the $x$-term: $(x_B - x_P)^2$ and taking the mean $\langle \cdot \rangle$ to get

$$\langle x^2 \rangle = \langle x_B^2 \rangle + 2\langle x_B x_P \rangle + \langle x_P^2 \rangle. \tag{S.16}$$

Assuming independence for both processes, we have $\langle x_B x_P \rangle = \langle x_B \rangle \langle x_P \rangle = 0$ and one gets

$$\langle x^2 \rangle = \langle x_B^2 \rangle + \langle x_P^2 \rangle. \tag{S.17}$$

For E. *Coli* we have $\langle x_B^2 \rangle = 2(v^2\tau/3)t$ where $v$ and $\tau$ is the average run velocity and time. For the phage $\langle x_P^2 \rangle = D_\alpha t^\alpha$ where $D_\alpha$ and $\alpha$ are the phage's diffusion coefficient and exponents. Thus, in three dimensions, we have

$$\langle r^2 \rangle = 2v^2\tau t + 6D_\alpha t^\alpha. \tag{S.18}$$

The time rate of change of the variance of $\boldsymbol{w}$ is the diffusivity $\mathfrak{D}(t)$ which is calculated as,

$$\mathfrak{D}(t) = \frac{1}{6}\frac{d}{dt}\langle r^2 \rangle = \frac{v^2\tau}{3} + \alpha D_\alpha t^{\alpha-1}. \tag{S.19}$$

Under the rest frame described above, an encounter model for the phage bacteria system can be treated exactly analogously to the flux of diffusing material to a perfectly absorbing sphere. So, if $P(r,t)$ is the concentration of phage as a function of distance $r$ from the center of the bacteria's spherical capture zone with radius $R_C$ then

$$\frac{\partial P}{\partial t} = \frac{1}{r^2}\frac{\partial}{\partial r}\left[r^2 \mathfrak{D}(t)\frac{\partial P}{\partial r}\right], \tag{S.20}$$

subject to the boundary conditions that $P(R_C,t) = 0$ (all phage are consumed at the surface of the bacteria's capture zone) and $P(r \to \infty, t) = P_\infty$, that at distances far from the bacteria the phage concentration approaches a constant background value. The initial conditions are $P(r,0) = 0$ for $r < R_C$ and $P(r,0) = P_\infty$ when $r \geq R_C$.

**Solution.** In Eq. (S.20), since $D(t)$ does not depend on $r$, it can be moved outside the differentials. The problem now more formally reads as

$$\frac{\partial P}{\partial t} = \frac{\mathfrak{D}(t)}{r^2}\frac{\partial}{\partial r}\left[r^2 \frac{\partial P}{\partial r}\right], \tag{S.21}$$

with specified boundary and initial conditions:

$$\begin{aligned} \text{B.C}: &\quad P(0,t) = 0 \\ \text{I.C}: &\quad P(r,0) = \begin{cases} 0, & \text{if } r \leq R_C, \\ P_\infty, & \text{if } r > R_C. \end{cases} \end{aligned}$$

To transform the 2-D problem into a more workable 1-D framework, we use the substitution $v(r,t) = P(r,t)r$. Now, taking first and second partial derivatives with respect to $r$ gives

$$\begin{aligned} \frac{\partial v}{\partial r} &= P + r\frac{\partial P}{\partial r} \\ \frac{\partial^2 v}{\partial r^2} &= \frac{\partial P}{\partial r} + \frac{\partial P}{\partial r} + r\frac{\partial^2 P}{\partial r^2} \\ &= 2\frac{\partial P}{\partial r} + r\frac{\partial^2 P}{\partial r^2}. \end{aligned}$$

So that

$$\frac{2}{r}\frac{\partial P}{\partial r} = \frac{1}{r}\frac{\partial^2 v}{\partial r^2} - \frac{\partial^2 P}{\partial r^2} \tag{S.22}$$

Taking the derivative of $v$ with respect to time in our substitution gives

$$\frac{\partial v}{\partial t} = r\frac{\partial P}{\partial z} \tag{S.23}$$

Expanding Eq. (S.21), and substituting Eq's (S.22) and (S.23) gives

$$\frac{\partial v}{\partial t} = \mathfrak{D}(t)\frac{\partial^2 v}{\partial r^2} \tag{S.24}$$

with specified boundary and initial conditions:

$$\begin{aligned} \text{B.C.} \quad & v(0,t) = 0 \\ \text{I.C.} \quad & v(r,0) = 0 \qquad (r \leq R_C), \\ & v(r,0) = P_\infty r \quad (r \geq R_C). \end{aligned}$$

A transform of variables; $z = r - R_C$ $(dz = dr)$, gives $v(r,t) \to w(z,t)$ and $z \geq 0$, if and only if $r \geq R_C$. For the initial conditions we have $w(z,t) = P_\infty z + P_\infty R_C$ for $z \geq 0$. The problem is now transformed into solving the diffusion equation on a semi-infinite interval $(z \geq 0)$. A standard way for solving the above problem, which can be found in most texts on partial differential equations, is to first introduce an odd extension of $w(z,t)$ as $-w(-z,t) = w(z,t)$ in the region $z < 0$. Next, using the method of Fourier transforms, one eventually arrives at the fundamental solution

$$w(z,t) = \frac{1}{\sqrt{4\pi\lambda(t)}}\int_{-\infty}^{\infty} w(\bar{z},0)\exp\left[-\frac{(z-\bar{z})^2}{4\lambda(t)}\right] d\bar{z}, \tag{S.25}$$

where we specially note,

$$\lambda(t) = \int_0^t \mathfrak{D}(t)\,dt.$$

While we have solved the problem, we need to transform the solution back into a usable form (into an expression involving the phage concentration). The physical solution of the phage concentration exists when $r \geq R_C$ which corresponds to $z \geq 0$. So, it makes sense to inspect our solution by separating the integrals in Eq. (S.25), to get

$$w(z,t) = \frac{1}{\sqrt{4\pi\lambda(t)}}\left(\int_{-\infty}^{0} w(\bar{z},0)\exp\left[-\frac{(z-\bar{z})^2}{4\lambda(t)}\right] d\bar{z} + \int_{0}^{\infty} w(\bar{z},0)\exp\left[-\frac{(z-\bar{z})^2}{4\lambda(t)}\right] d\bar{z}\right),$$

So that upon letting $z \to -z$ in the negative integral and applying the oddness of $w$ gives

$$w(z,t) = \frac{1}{\sqrt{4\pi\lambda(t)}} \int_0^\infty w(\bar{z},0) \left\{ \exp\left[ -\frac{(z-\bar{z})^2}{4\lambda(t)} \right] - \exp\left[ -\frac{(z+\bar{z})^2}{4\lambda(t)} \right] \right\} d\bar{z}.$$

Noting $v(\bar{z},0) = P_\infty \bar{z} + P_\infty R_C$ for $\bar{z} \geq 0$ and back-transforming to $r$ ($z = r - R_C$, $\bar{z} = \bar{r} - R_C$) gives

$$v(r,t) = \frac{1}{\sqrt{4\pi\lambda(t)}} \int_{R_C}^\infty P_\infty \bar{r} \left\{ \exp\left[ -\frac{(r-\bar{r})^2}{4\lambda(t)} \right] - \exp\left[ -\frac{(r+\bar{r}-2R_C)^2}{4\lambda(t)} \right] \right\} d\bar{r},$$

$$= \frac{1}{\sqrt{4\pi\lambda(t)}} \int_{R_C}^\infty P_\infty \bar{r} \left\{ \exp\left[ -\frac{(r-\bar{r})^2}{4\lambda(t)} \right] - \exp\left[ -\frac{(r+\bar{r}-2R_C)^2}{4\lambda(t)} \right] \right\} d\bar{r}.$$

More compactly we can write

$$v(r,t) = \frac{1}{\sqrt{4\pi\lambda(t)}} I_1. \tag{S.26}$$

Expanding the integral $I_1$ we have,

$$I_1 = \int_{R_C}^\infty P_\infty \bar{r} \left\{ \exp\left[ -\frac{(r-\bar{r})^2}{4\lambda(t)} \right] - \exp\left[ -\frac{(r+\bar{r}-2R_C)^2}{4\lambda(t)} \right] \right\} d\bar{r},$$

$$= \int_{R_C}^\infty P_\infty \bar{r} \exp\left[ -\frac{(r-\bar{r})^2}{4\lambda(t)} \right] d\bar{r} - \int_{R_C}^\infty P_\infty \bar{r} \exp\left[ -\frac{(r+\bar{r}-2R_C)^2}{4\lambda(t)} \right] d\bar{r}.$$

Letting $\xi = (\bar{r} - r)/\sqrt{4\lambda(t)}$ so that $\bar{r} = \sqrt{4\lambda(t)}\xi + r$ in the first integral and $\xi = \left(\bar{r} + r - 2R_{\text{cap}}\right)/\sqrt{4\lambda(t)}$ so that $\bar{r} = \sqrt{4\lambda(t)}\xi - r + 2R_{\text{cap}}$ in the second integral we get

$$I_1 = P_\infty 4\lambda(t) \int_{(R_C - r)/\sqrt{4\lambda(t)}}^\infty \xi \exp\left(\xi^2\right) d\xi + \sqrt{4\lambda(t)} P_\infty r \int_{(R_C - r)/\sqrt{4\lambda(t)}}^\infty \exp\left(\xi^2\right) d\xi,$$

$$- P_\infty 4\lambda(t) \int_{-(R_C - r)/\sqrt{4\lambda(t)}}^\infty \xi \exp\left(\xi^2\right) d\xi + \sqrt{4\lambda(t)} P_\infty r \int_{-(R_C - r)/\sqrt{4\lambda(t)}}^\infty \exp\left(\xi^2\right) d\xi$$

$$- 2\sqrt{4\lambda(t)} P_\infty R_C \int_{-(R_C - r)/\sqrt{4\lambda(t)}}^\infty \exp\left(\xi^2\right) d\xi$$

$$= -P_\infty 4\lambda(t) \int_{-\left(R_{\text{cap}} - r\right)/\sqrt{4\lambda(t)}}^{\left(R_{\text{cap}} - r\right)/\sqrt{4\lambda(t)}} \xi \exp\left(\xi^2\right) d\xi + 2\sqrt{4\lambda(t)} P_\infty r \int_0^\infty \exp\left(\xi^2\right) d\xi$$

$$- 2\sqrt{4\lambda(t)} P_\infty R_C \int_{-(R_C - r)/\sqrt{4\lambda(t)}}^\infty \exp\left(\xi^2\right) d\xi.$$

Due to the oddness of $\xi \exp\left(\xi^2\right)$ the first integral evaluates to zero. Also, $\int_0^\infty \exp\left(\xi^2\right) d\xi = \sqrt{\pi}/2$. So, we are left with

$$
\begin{aligned}
I_1 &= \sqrt{4\pi\lambda(t)}P_\infty r - 2\sqrt{4\lambda(t)}P_\infty R_C \int_{-(R_C-r)/\sqrt{4\lambda(t)}}^{\infty} \exp\left(\xi^2\right) d\xi \\
&= \sqrt{4\pi\lambda(t)}P_\infty r - 2\sqrt{4\lambda(t)}P_\infty R_C \left(\int_{-(R_C-r)/\sqrt{4\lambda(t)}}^{0} \exp\left(\xi^2\right) d\xi + \int_0^\infty \exp\left(\xi^2\right) d\xi\right) \\
&= \sqrt{4\pi\lambda(t)}P_\infty r - 2\sqrt{4\lambda(t)}P_\infty R_C \int_0^\infty \exp\left(\xi^2\right) d\xi - 2\sqrt{4\lambda(t)}P_\infty R_C \int_{-(R_C-r)/\sqrt{4\lambda(t)}}^{0} \exp\left(\xi^2\right) d\xi \\
&= \sqrt{4\pi\lambda(t)}P_\infty r - \sqrt{4\pi\lambda(t)}P_\infty R_C - 2\sqrt{4\lambda(t)}P_\infty R_C \int_0^{(R_C-r)/\sqrt{4\lambda(t)}} \exp\left(\xi^2\right) d\xi \\
&= \sqrt{4\pi\lambda(t)}P_\infty (r - R_C) - \sqrt{4\pi\lambda(t)}\,\mathrm{erf}\left(\frac{R_C - r}{\sqrt{4\lambda(t)}}\right)
\end{aligned}
$$

Thus,

$$v(r,t) = P_\infty (r - R_C) + P_\infty R_C \,\mathrm{erf}\left(\frac{R_C - r}{\sqrt{4\lambda(t)}}\right)$$

Finally, since $v(r,t) = P(r,t)r$ we have

$$P(r,t) = P_\infty \left(1 - \frac{R_C}{r}\right) + \left(\frac{P_\infty R_C}{r}\right) \mathrm{erf}\left(\frac{R_C - r}{\sqrt{4\lambda(t)}}\right)$$

The phage flux (encounter rate) $\mathscr{J}(t)$ is then given by

$$\mathscr{J}(t) = \oint \mathfrak{D}(t) \frac{\partial P}{\partial r} \hat{r} \cdot d\vec{s},$$

for which we can evaluate at $r = R_C$ to get the time dependent solution,

$$\mathscr{J}(t) = 4\pi P_\infty R_C \mathfrak{D}(t) \left(1 + \sqrt{\frac{R_C^2}{\pi\lambda(t)}}\right). \tag{S.27}$$

This solution shows the phage encountered varies $\lambda(t)^{1/2}$ for small $t$ and for $t \gg 1$ asymptotically tends to the steady state encounter rate

$$\mathscr{J}_{\text{diff}} = 4\pi P_\infty R_C \mathfrak{D}, \tag{S.28}$$

where $\mathfrak{D} \approx v_B^2 \langle \tau_R \rangle / 3$ for $\alpha < 1$ and $\mathfrak{D} \approx v_B^2 \langle \tau_R \rangle / 3 + D$ for $\alpha = 1$. Upon dividing by $P_\infty$ gives the encounter kernel

$$\beta_{\text{diff}} = 4\pi R_C \mathfrak{D}, \tag{S.29}$$

which is essentially the maximum clearance rate of the phage.
